# Clinical, Electrocardiographic, Biochemical, and Echocardiographic Markers for Diagnosing Cardiac Dysfunction in Neonates with Hypoxic–Ischemic Encephalopathy: A Narrative Review

**DOI:** 10.3390/children13081050

**Published:** 2026-08-06

**Authors:** Solomon Tatagiri, Belinda Chan, Yogen Singh

**Affiliations:** Neonatology Division, Department of Pediatrics, University of California at Davis, Sacramento, CA 95817, USA; sptatagiri@health.ucdavis.edu (S.T.); belchan@health.ucdavis.edu (B.C.)

**Keywords:** hypoxic–ischemic encephalopathy, neonatal encephalopathy, cardiovascular dysfunction, echocardiography, cardiac biomarkers, right ventricular dysfunction, pulmonary hypertension, therapeutic hypothermia

## Abstract

**Highlights:**

**What are the main findings?**

• Cardiovascular dysfunction is highly prevalent in moderate-to-severe HIE, identified in up to 70–90% of neonates when multimodal assessment is used, and it independently predicts both mortality and adverse neurodevelopmental outcomes.
• Advanced echocardiographic techniques, including tissue Doppler imaging and speckle-tracking strain analysis, detect myocardial dysfunction more sensitively than conventional measures such as ejection fraction and fractional shortening.
• Right ventricular dysfunction is consistently associated with adverse clinical outcomes.

**What are the implications of the main findings?**

• Hemodynamic management should move away from reliance on conventional clinical markers, such as blood pressure, towards a multimodal, physiology-based approach combining cardiac biomarkers, electrocardiography, near-infrared spectroscopy (NIRS) and targeted neonatal echocardiography (TnECHO).
• Standardized definitions, validated biomarker thresholds, and prospective multicenter interventional trials are needed to establish whether physiology-guided cardiovascular care improves neurological outcomes.

**Abstract:**

**Background/Objectives:** Hypoxic–ischemic encephalopathy (HIE) remains a leading cause of neonatal death and long-term neurodisability. Cardiovascular dysfunction commonly accompanies HIE and influences neurological recovery, yet it is difficult to recognize because conventional clinical signs correlate poorly with the myocardial function. This review summarizes the current evidence on cardiac dysfunction in neonates with HIE, with particular attention to the diagnostic tools used to evaluate cardiovascular function and the clinical outcomes associated with myocardial injury. **Methods:** We conducted a narrative review informed by a systematic, reproducible search of four databases (PubMed, Embase, Cochrane Library, and Web of Science) from 2000 to 2026, using a Population–Concept–Context framework to identify studies of term and near-term neonates (≥35 weeks gestation) with HIE or perinatal asphyxia reporting a measure of cardiac dysfunction (electrocardiographic, biomarker, echocardiographic, or clinical hemodynamic). Records were screened and selected using Covidence systematic review software; eligible studies were original, primary-data reports with a minimum sample size of 20 neonates. Findings were synthesized narratively and grouped thematically by diagnostic modality and clinical outcome. **Results:** Eighty-one studies met the pre-specified eligibility criteria following a systematic multi-database search and structured screening process. As a subset of included studies are secondary analyses or overlapping cohorts, an aggregate patient count is not reported. Reported prevalence of cardiac dysfunction varied widely with the definition applied, reaching 70–90% when multimodal assessment was used. Blood pressure, heart rate, urine output, and lactate were inconsistently reliable indicators of myocardial function, and ejection fraction and fractional shortening often remained normal despite injury. Tissue Doppler imaging and speckle-tracking strain appeared to be more sensitive, with impaired left ventricular global longitudinal strain associated with brain injury. The right ventricle was more commonly and severely affected, with reduced TAPSE, RV fractional area change, and RV strain associated with death or MRI-defined injury. Each additional 24 h of inotropic support was associated with increased odds of adverse short- and long-term outcomes by 14% and 12%, respectively. **Conclusions:** Cardiac dysfunction is common and prognostically important but remains underrecognized by the routine clinical assessment and cardiovascular monitoring. Multimodal monitoring centered on TnECHO, near infra-red spectroscopy, and specific biomarkers may enable earlier, physiology-based management.

## 1. Introduction

Hypoxic–ischemic encephalopathy (HIE) remains one of the major causes of neonatal death and long-term neurodisability worldwide, with an estimated incidence of 1 to 2 per 1000 live births in high-income countries and much higher rates reported across low- and middle-income countries [1,2]. Overall, HIE is responsible for approximately 22–23 percent of all neonatal deaths globally, and among survivors, a significant proportion develop long-term neurodevelopmental complications, including cerebral palsy, epilepsy, and cognitive and developmental delays [3,4]. The primary cause of initial injury is hypoxia, which is typically triggered by an acute sentinel event, such as cord prolapse, placental abruption, or shoulder dystocia. It interrupts oxygen delivery to the brain which initiates a well-characterized cascade of neuronal injury events [5]. Therapeutic hypothermia (TH) remains the only approved neuroprotective treatment for moderate to severe HIE, where the neonate is targeted to a core temperature of 33–34 °C for 72 h. It has been shown to reduce the composite outcome of death or major neurodisability by 25 to 30 percent in randomized controlled trials [6,7]. Despite optimal TH, up to 40 percent of treated neonates continue to die or survive with significant neurological sequelae [8,9].

**Figure 1:**
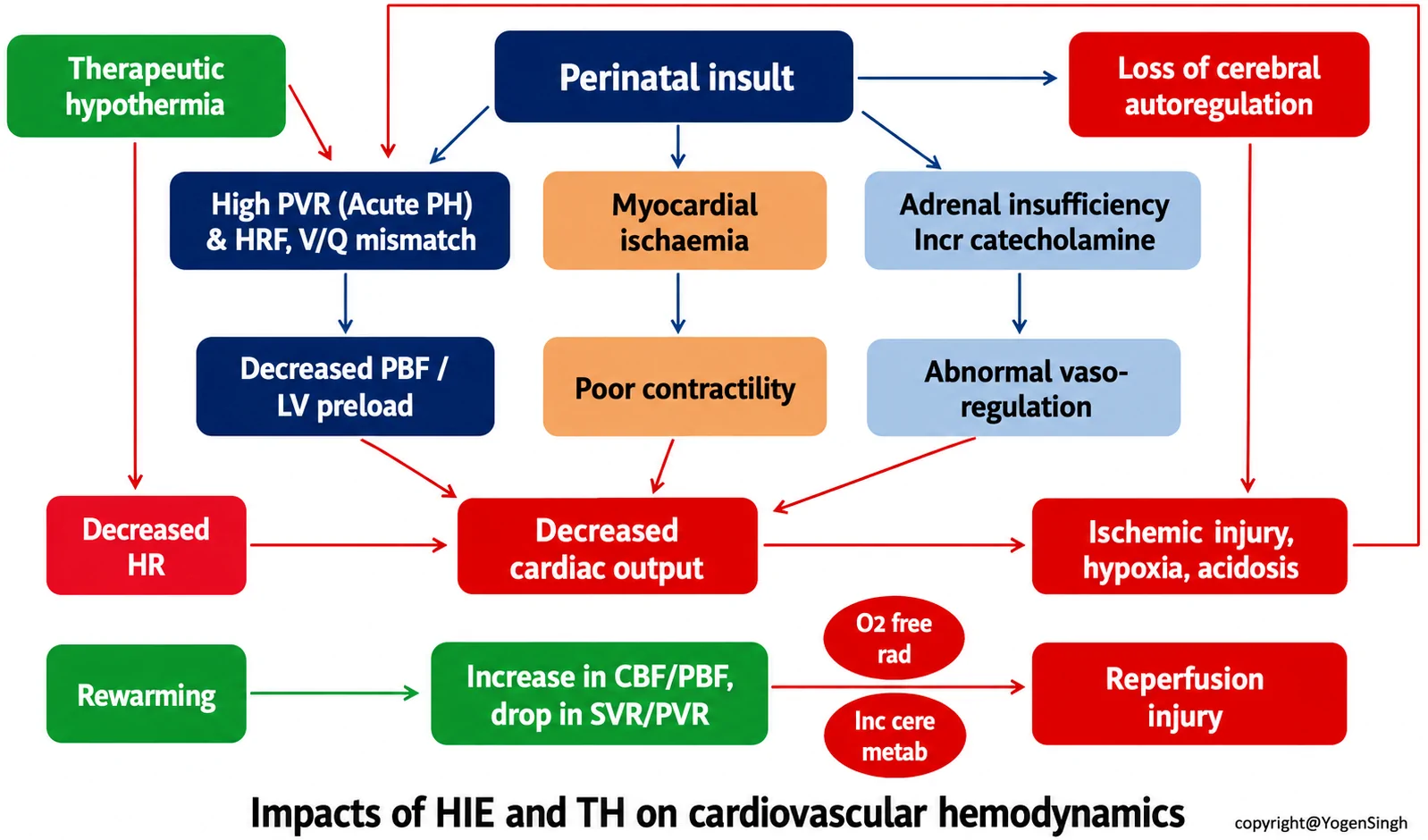
Pathophysiology of hemodynamics in HIE—illustrating mechanism and impacts of HIE and therapeutic hypothermia on cardiovascular hemodynamics. The figure illustrates the sequence of cardiovascular events initiated by a sentinel hypoxic event at or around birth. Abbreviations: HIE, hypoxic–ischemic encephalopathy; LV, left ventricle; PVR, pulmonary vascular resistance; PH, Pulmonary hypertension; HRF, Hypoxemic respiratory failure; V/Q mismatch, Ventilation Perfusion mismatch; PBF, Pulmonary blood flow; CBF, Cerebral blood flow; SVR, Systemic vascular resistance; HR, Heart rate; TH, therapeutic hypothermia; Inc cere metab, Increased cerebral metabolism.

**Figure 2:**
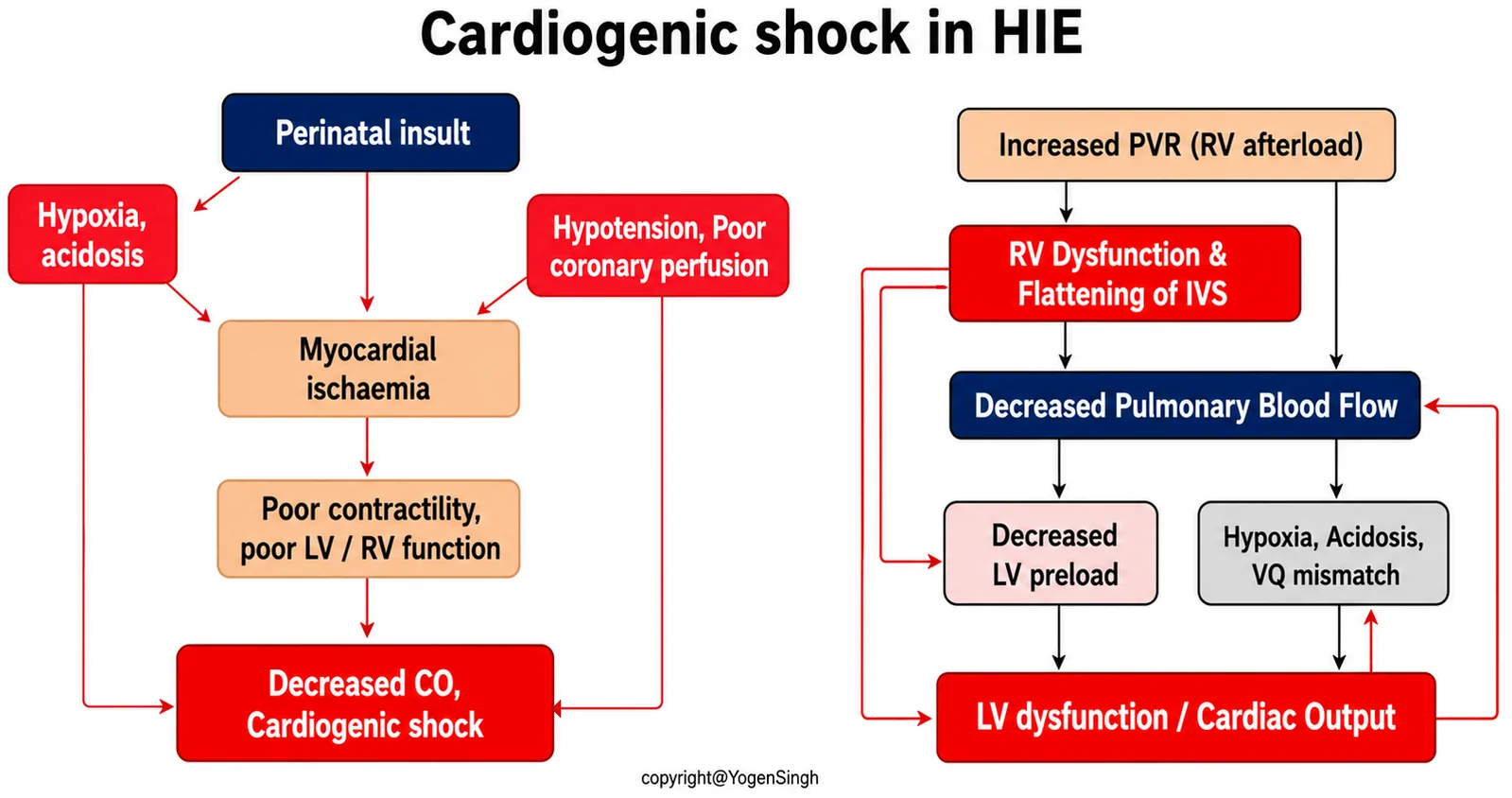
Pathophysiology of cardiogenic shock in infants with HIE undergoing therapeutic hypothermia. Abbreviations: CO, cardiac output; RV, right ventricle; IVS, interventricular septum; see Figure 1 for other abbreviations.

It has long been recognized that HIE causes multiorgan injuries extending well beyond the brain. Martin-Ancel et al. documented that perinatal asphyxia produces multiorgan dysfunction, with cardiac involvement identified in approximately 29 percent of the neonates [10]. Shah et al. reported that all neonates with moderate-to-severe HIE demonstrate dysfunction in at least one extracranial organ system, with cardiovascular dysfunction noted in up to 62 percent [11]. Myocardial injury in neonatal HIE occurs in two phases: an initial hypoxic phase characterized by intracellular acidosis and calcium overload, followed by reperfusion injury in which restoration of blood flow generates reactive oxygen species that cause further cardiomyocyte damage [12,13]. During the acute insult, the diving reflex redistributes cardiac output toward vital organs; namely, the brain, heart and adrenals. However, persistent hypoxia progressively depletes myocardial energy stores and impairs coronary perfusion, resulting in worsening myocardial dysfunction [14,15]. The right ventricle (RV) is particularly vulnerable due to its crescent-shaped geometry, higher wall stress, and dominant role in transitional circulation [16]. In the setting of hypoxia and acidosis, failure of postnatal pulmonary vasodilation leads to persistent pulmonary hypertension (PPHN), imposing sustained pressure overload on the RV that is further amplified with application of TH [17,18]. Pathophysiology of hemodynamics instability has been illustrated in Figure 1 and Figure 2.

Among the organ systems affected in HIE, the cardiovascular system plays a critical role. It is vulnerable to hypoxic–ischemic injury at birth, yet postnatal neurological recovery from HIE depends on adequate cardiovascular function and perfusion. Therefore, early recognition of hemodynamic instability is essential to guide physiology-based management and optimize cardiovascular support.

### Rationale for the Review and Objectives

Evaluation of cardiovascular dysfunction in neonates with HIE remains challenging [19]. Conventional parameters such as heart rate, arterial blood pressure, capillary refill time, urine output, and serum lactate are routinely used to guide hemodynamic management, yet they often fail to reflect the true underlying physiology and they all are the proxy parameters of cardiovascular health [20,21]. These parameters are good trending markers, but they lack sensitivity and specificity. Elevated serum lactate may stem from perinatal poor tissue perfusion rather than current myocardial dysfunction, and reduced urine output may reflect hypoxic kidney injury rather than impaired systemic perfusion. Blood pressure is equally unreliable; a multinational survey by Giesinger et al. across 71 NICUs in North America revealed that clinicians vary in defining neonatal hypotension in neonates with HIE and thresholds for intervention [19]. Blood pressure poorly correlates with cardiac function, and more worryingly, it may be maintained in normal range despite very low cardiac output due to increased systemic vascular resistance from TH or medications. Hence, blood pressure is a late and unreliable sign of hemodynamic instability in infants with HIE undergoing TH [22,23]. A study demonstrated that arterial blood pressure frequently normalizes following inotrope administration in neonates with HIE on therapeutic hypothermia, even as RV dysfunction persists, creating a false sense of hemodynamic recovery [24].

Given these limitations, over the last two decades considerable research efforts have focused on improving the early recognition and diagnosis of hemodynamic instability and refining the management strategies. This narrative review aims to summarize the current evidence on cardiac dysfunction in neonates with HIE, encompassing the diagnostic tools used to evaluate cardiovascular function and the clinical outcomes associated with myocardial injury in this population.

## 2. Materials and Methods

**Figure 3:**
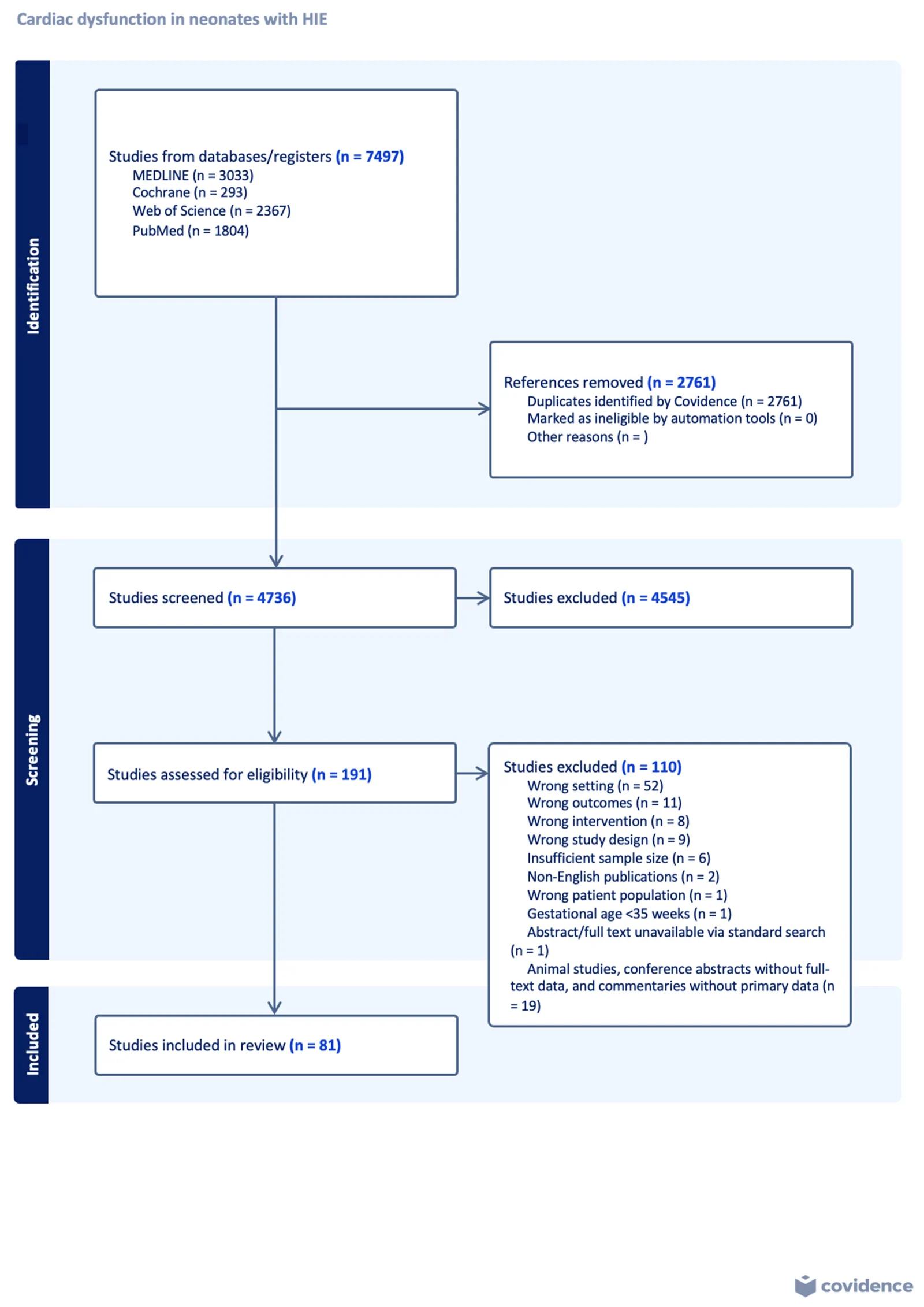
PRISMA flow diagram of study identification and selection for a narrative review of cardiac dysfunction in neonates with hypoxic–ischemic encephalopathy.

This manuscript is a narrative review of the published literature on cardiac dysfunction in neonates with HIE. The review question, eligibility criteria, and search strategy were structured using a Population–Concept–Context (PCC) framework and a systematic, multi-database search and screening process (detailed below); however, the synthesis of findings remains narrative rather than following full PRISMA Scoping Review (PRISMA-ScR) reporting, given the substantial heterogeneity in study design, outcome definitions, and assessment. We therefore describe this as a narrative review through a systematic, reproducible search and screening methodology, and report the study selection process using a PRISMA flow diagram (Figure 3) for transparency. No prospective protocol for this review was registered. The aims and objectives were to map the available evidence, identify the range of diagnostic tools and outcomes studied, and delineate gaps that should inform future research priorities.

### 2.1. Eligibility Criteria

Inclusion and exclusion criteria were defined using the PCC framework:

• Population: Term and near-term neonates (gestational age ≥ 35 weeks) with a diagnosis of HIE or perinatal asphyxia.
• Concept: Any form of cardiac dysfunction including, but not limited to, echocardiographic abnormalities (systolic or diastolic ventricular dysfunction, pulmonary hypertension, reduced cardiac output); elevation of cardiac biomarkers such as Cardiac Troponin I (cTnI), Cardiac Troponin T (cTnT), Creatine Kinase (CK), Creatine Kinase-MB (CK-MB), Brain Natriuretic Peptide (BNP), and N-terminal pro-B-type natriuretic peptide (NT-proBNP); electrocardiographic changes (ST segment changes, T-wave abnormalities, QTc prolongation, arrhythmia); clinical hemodynamic instability (hypotension, inotrope requirement, shock); or indirect measures such as near-infrared spectroscopy (NIRS) cerebral oxygenation and lactate.
• Context: Neonatal intensive care unit (NICU) setting; studies addressing the periasphyxial period, the period of TH, rewarming, or post-NICU follow-up.
• Original studies reporting primary patient-level data, with a minimum sample size of 20 neonates.

### 2.2. Exclusion Criteria

• Neonates with congenital heart disease (CHD), chromosomal abnormalities, or major malformation;
• Gestational age < 35 weeks;
• Non-English publications;
• Wrong setting/context (e.g., antenatal or fetal-only assessment, non-NICU populations, or timing outside the periasphyxial/TH/rewarming/follow-up window);
• Wrong concept/outcome (no cardiac dysfunction related outcome reported, as defined above);
• Wrong population/exposure (cardiac dysfunction studied in a NICU population not centered on HIE or perinatal asphyxia);
• Study design ineligible for primary evidence synthesis (narrative or systematic reviews, editorials, correspondence, or trial registrations without reported results);
• Insufficient sample size (fewer than 20 neonates);
• Abstract/full text unavailable via standard search;
• Animal studies, conference abstracts without full-text data, and commentaries without primary data.

### 2.3. Information Sources and Search Strategy

Searches were conducted across four databases—PubMed, MEDLINE, Cochrane Library, and Web of Science—from 2000 (the earliest indexed report of electrocardiographic myocardial injury grading in asphyxiated neonates, the Jedeikin criteria study in 1983 is included as it served as a landmark study for EKG characterization and interpretation in neonates with HIE) to 2026.

Search terms were combined using Boolean operators across three concept blocks: (1) population terms (“hypoxic ischemic encephalopathy”, “perinatal asphyxia”, “birth asphyxia”, “neonatal encephalopathy”, “intrapartum asphyxia”); (2) concept terms (“cardiac dysfunction”, “myocardial dysfunction”, “myocardial injury”, “ventricular dysfunction”, “echocardiography”, “troponin”, “cardiac biomarker”, “pulmonary hypertension”, “hemodynamics”, “inotrope”, “cardiac output”, “right/left ventricle”, “myocardial strain”, “speckle tracking”, “tissue Doppler”, “therapeutic hypothermia”); and (3) population-age terms (“neonate”, “newborn”, “infant”), joined with ‘AND’. Searches were limited to human studies published in English.

### 2.4. Study Selection and Data Charting

Records identified across the four databases (n = 7497: PubMed 1804; MEDLINE 3033; Cochrane 293; Web of Science 2367) were imported into Covidence systematic review software and deduplicated (2761 duplicates removed), leaving 4736 unique records for title and abstract screening. Title and abstract screening and full-text review were performed against the eligibility criteria above independently in duplicate by two reviewers. Of the 4736 records screened, 4545 were excluded, and 191 were sought for and obtained in full text. Following full-text assessment, 110 studies were excluded, as mentioned in Figure 3. This yielded a final set of 81 studies. The complete study identification and selection process is presented as a PRISMA flow diagram (Figure 3) [25]. Data charting was performed using a standardized extraction form capturing study design, country, sample size, gestational age, HIE severity grading, cooling protocol, cardiac assessment tools used, timing of assessment, definitions of cardiac dysfunction applied, and clinical outcomes reported. No formal quality or risk-of-bias appraisal tool (e.g., the Newcastle–Ottawa Scale) was applied to individual studies; this is stated explicitly as a limitation.

### 2.5. Synthesis Approach

Given the considerable heterogeneity in study design, patient populations, diagnostic definitions, and outcome measures across the published literature, a formal meta-analysis was not performed. Instead, a narrative synthesis approach was used, in which findings were grouped thematically by diagnostic modality (electrocardiography, cardiac biomarkers, conventional echocardiography, advanced echocardiographic techniques) and by clinical outcome domain.

Non-primary sources (narrative or systematic reviews, editorials, and correspondence) identified during screening were used to contextualize the discussion but were not included in the primary evidence synthesis or summary tables. Companion publications evaluating different outcomes but derived from the same patient cohort were retained as they reported unique outcome measures.

Given the predominance of observational, retrospective, and single-center study designs among included primary studies, findings are reported as associations rather than as evidence of prediction or causality, unless a specific study’s design explicitly supports stronger inference.

## 3. Results

### 3.1. Overview of Included Studies and Patient Population

Eighty-one studies met the final inclusion criteria following the systematic search, screening, and eligibility process described in the Section 2. An aggregate neonate sample size is not reported here because several included studies are secondary analyses of the overlapping cohorts. Studies included prospective observational cohorts, retrospective cohort or case–control studies, and secondary analyses of randomized controlled trials.

### 3.2. Epidemiology of Cardiac Dysfunction in HIE

The reported prevalence of cardiac dysfunction across studies varied substantially, most likely due to differences in how it was defined in the diagnostic modalities used. Martín-Ancel et al. (1995) and Rasheed et al. (2024) reported the prevalence of cardiac dysfunction to be around 29% [10,26], while Bhasin et al. (2019) and Hochwald O et al. (2014) reported the prevalence to be around 100%, with predominantly neonates with moderate-to-severe HIE [27,28]. Additionally, one prospective cohort study identified significant day-of-life 2 echocardiographic ventricular dysfunction in approximately 29% of TH neonates [29]. Other studies, utilizing broader definitions that include electrocardiographic signs of ischemia and biochemical markers, report cardiovascular involvement in nearly 70–90% of patients [30,31,32]. The incidence of acute pulmonary hypertension (aPH) or PPHN is similarly elevated in this population, affecting between 13% and 28% of neonates with HIE receiving TH [33,34,35,36], and varies based on the severity of HIE. The prevalence of hemodynamic instability requiring inotropic support ranges from 27% to 75% across major clinical trials [37,38,39,40]. The studies that included a higher proportion of neonates with severe HIE and pulmonary hypertension reported inotrope use rates as high as 87–96% [33,36,41]. This high burden of cardiovascular instability represents a critical need for robust diagnostic modalities and targeted interventions, as cardiac dysfunction serves as an independent predictor of mortality and adverse neurodevelopmental outcomes [16,20].

### 3.3. Diagnostic Modalities: Electrocardiography (EKG/ECG)

Several studies have evaluated EKG changes in neonates with HIE. The seminal work by Jedeikin et al. (1983) [42] described characteristic T-wave abnormalities in stressed neonates and proposed a grading system to classify the severity of myocardial ischemia, known as the Jedeikin criteria (Table 1). It grades ischemic changes from I to IV based on T-wave morphology, ST-segment shifts, and the presence of pathological Q-waves [42]. This classification has subsequently been widely adopted in studies assessing myocardial injury following perinatal asphyxia.

Grade I and II changes are highly prevalent but lack specificity, as these changes are frequently seen in EKG of healthy neonates [42,43,44]. Conversely, Grade III and IV changes are more specific markers of severe myocardial injury [30,43]. In severe HIE, EKG patterns of acute inferior myocardial infarction with deep Q waves have been documented and are strongly correlated with early mortality [45]. EKG monitoring in neonates with HIE undergoing TH for HIE commonly demonstrates sinus bradycardia and QTc prolongation findings that are more reflective of the effects of cooling itself than of the underlying hypoxic–ischemic injury [46]. Overall, EKG is best used in combination with biomarkers and echocardiography to assess cardiac dysfunction, particularly in the early phase of HIE or in milder cases [47,48].

Table 2 summarizes key studies evaluating EKG changes in neonates with HIE, along with their main conclusions and limitations.

### 3.4. Diagnostic Modalities: Cardiac Biomarkers

Cardiac biochemical markers provide essential insight into subclinical myocardial injury. Numerous studies assessed Troponins (cTnI or cTnT), Total CK, CK-MB, BNP/NT-proBNP, and novel biomarkers in over 1500 neonates (Table 3).

Cardiac troponins (cTnT and cTnI) are parts of the structural proteins of the contractile apparatus of cardiac muscle and hence form a marker for myocardial injury [87,88]. They typically peak at around 12–24 h [31,61]. CK-MB, on the other hand, is released due to cell membrane damage from either the cardiac muscle or skeletal muscle cells. Troponins T and I are considered superior over CK-MB due to their resistance to confounding factors such as skeletal muscle trauma during delivery [34,57,74].

#### 3.4.1. Cardiac Troponin T (cTnT)

No uniform threshold for cTnT exists to define myocardial injury in neonates with HIE, reflecting the considerable heterogeneity across published studies (Table 3) [31,34,44]. While there is broad agreement that higher cTnT levels correlate significantly with HIE severity and mortality, the cutoff values used to define myocardial injury or predict outcomes vary widely across studies. For instance, Boo NY et al. identified a threshold of >0.1 µg/L as clinically relevant for diagnosing myocardial injury [31], while Joseph S et al. demonstrated that a higher threshold of >0.25 µg/L predicted mortality, with sensitivity of 83.9% and specificity of 96.6% [57].

#### 3.4.2. Cardiac Troponin I (cTnI)

Concentration of cTnI also demonstrates a positive correlation with HIE severity [74]. A level < 0.15 ng/L at 24 h of age in neonates undergoing TH has been identified as a predictor of favorable neurodevelopmental outcomes at 18–22 months [61].

#### 3.4.3. Creatine Kinase-MB (CK-MB)

Multiple studies have associated elevated CK-MB with HIE severity and mortality [28,30,43], but CK-MB is less specific because it can be influenced by gestational age, birth weight, and mode of delivery (with higher levels noted in vaginal vs. cesarean births) [57]. Hence, Barberi et al. report that elevated CK-MB (>154 IU/L) on the second day of life, in addition to concerning EKG and echocardiographic findings, is associated with a poor clinical prognosis [43]. The most recent study by Moore et al. (2025) evaluated the relationship between serial cardiac biomarkers and echocardiographic RV function in 56 neonates with HIE [85]. A total CK threshold of 1961 U/L identified reduced Tricuspid Annular Plane Systolic Excursion (TAPSE) associated with decreased RV function with high sensitivity (93%) but moderate specificity (55%), while a cTnI threshold of 91 ng/L (0.091 µg/L) had a sensitivity of 79% and specificity of 74%.

### 3.5. Diagnostic Modalities: Conventional Echocardiography and Advanced Echocardiographic Techniques (Tissue Doppler Imaging and Speckle Tracking)

Echocardiography plays a central role in evaluating cardiac function in HIE. Table 4 outlines the parameters most commonly used across studies, while Table 5 summarizes the key echocardiographic findings associated with HIE.

#### 3.5.1. Cardiac Output

Echocardiographic metrics demonstrate reduced systemic flow in neonates with moderate-to-severe HIE. Left ventricular output (LVO) and right ventricular output (RVO) are consistently reduced during the first 24–48 h of life, often falling below 150 mL/kg/min [91,95,113]. While LVO and RVO are markedly depressed during TH, they typically demonstrate a steady recovery following rewarming [93,114]. Hochwald et al. demonstrated that superior vena cava (SVC) flow, a surrogate marker of cerebral venous return and brain perfusion, was preserved despite reductions in systemic cardiac output, suggesting preferential redistribution of blood flow to the brain [27]. Under normal conditions, cerebrovascular autoregulation maintains relatively stable cerebral perfusion across a range of systemic blood pressures, buffering the brain from hemodynamic fluctuations. However, maintained or elevated cerebral flow does not necessarily confer benefit; indeed, an inappropriately elevated SVC flow velocity without intact autoregulation was associated with MRI-confirmed brain injury [27]. Van Bel & Walther provided direct evidence of impaired autoregulation in HIE, demonstrating that cerebral blood flow velocity varied passively with changes in blood pressure rather than remaining stable [41]. When this protective mechanism is lost, the cerebral vasculature becomes pressure-passive, rendering the brain vulnerable to both hypoperfusion during hypotension and hyperemia during hypertension, each capable of compounding the underlying ischemic injury.

#### 3.5.2. Global Systolic Function

Interestingly, in some infants with raised biochemical markers suggestive of cardiovascular injury, global markers of systolic function such as ejection fraction (EF) and fractional shortening (FS) often remain within normal limits or are not statistically significant, even in neonates with severe HIE and low systemic flow [29,96,115]. This suggests that conventional measures like FS may lack sensitivity for detecting subtle myocardial dysfunction.

Across studies, tissue Doppler imaging (TDI) consistently detected myocardial dysfunction in neonates with HIE that was frequently missed by conventional echocardiography. Even when EF and FS remained within normal limits, TDI parameters, including reduced peak systolic velocity (Sm) and an elevated Tei index reflecting combined systolic and diastolic dysfunction, revealed persistent, biventricular impairment [100,101]. TDI further demonstrated reduced strain and strain rate in HIE neonates compared with controls, indicating diminished intrinsic myocardial deformation and contractile velocity at the myofiber level [102]. Notably, these strain abnormalities were present irrespective of TH exposure [103]. TDI also identified early diastolic dysfunction, characterized by prolonged isovolumic relaxation time (IVRT′), reduced right ventricular early diastolic velocity (e′), and an increased myocardial performance index (MPI′) [35]. Importantly, these abnormalities generally resolved after rewarming or within the first few days of life [102,103].

Speckle-tracking echocardiography (STE) is another advanced echocardiographic technology that can detect impaired LV and RV global longitudinal strain (GLS) and strain rate (SR) in HIE neonates with preserved EF and FS [38,110]. Longitudinal strain is particularly sensitive to subtle myocardial injury, as it assesses the function of subendocardial fibers, which are highly susceptible to ischemia [108,116]. Sehgal et al. reported that LV GLS was severely impaired in asphyxiated neonates. Neonates who died had lower GLS than survivors (−9.7% vs. −12.8%, *p* = 0.02), showing the prognostic value of the measurement [108]. Furthermore, systolic strain rate is identified as a more load-independent measure of contractility than either cardiac output or strain, as GLS is not influenced by the increased systemic vascular resistance (afterload) induced by TH [47,117]. Speckle-tracking can also reveal abnormalities in more advanced mechanics, including reduced rotational dynamics (twist, torsion, and untwist), indicating global myocardial dysfunction beyond simple systolic measures [109]. These deformation abnormalities normalized over time [115].

#### 3.5.3. Right Ventricular (RV) Performance

Elevated pulmonary vascular resistance (PVR) is common in neonates with HIE, often driven by underlying perinatal factors such as asphyxia, acidosis, and associated pulmonary pathology; TH may further increase pulmonary vascular tone. As a result, RV performance is frequently under increased afterload, placing RV performance under significant stress. Echocardiographic evidence of elevated PVR in those patients includes increased systolic eccentricity indices (sEI), right-to-left or bidirectional shunting across the ductus arteriosus or PFO, and increased tricuspid regurgitation (TR) jet velocity, which is used to calculate RV systolic pressure [47].

TAPSE is the primary conventional marker for RV longitudinal and global function, which are frequently reduced in neonates with HIE [16,118]. At 24 h of life, both parameters were independently associated with adverse outcome (death and/or abnormal MRI) [16]. In a subsequent study, Giesinger et al. reported that a TAPSE value < 6 mm predicted adverse neurodevelopmental outcomes, with high sensitivity (91%) and specificity (81%) [119]. Rodriguez MJ et al. reported similar findings of reduced TAPSE and RV stroke volume in neonates with moderate-to-severe HIE undergoing TH. The authors suggested that the RV failure may contribute to impaired brain perfusion and subsequent brain injury [96].

While TAPSE and fractional area change (FAC) remain clinically useful, advanced deformation imaging, such as RV Free Wall Strain (FWS) and 4-chamber strain, provides a more sensitive and detailed assessment of regional myocardial function and contractility [38]. Jin et al. (2025) report that impaired RV strain values correlate more strongly with decreased survival to discharge and increased need for inotropic support than traditional markers like TAPSE or FAC [38].

#### 3.5.4. Pulmonary Hemodynamics and Afterload

Given the increased RV afterload, assessing pulmonary hemodynamics in detail helps better define the severity and impact of elevated PVR. While estimating Right Ventricular Systolic Pressure (RVSP) via tricuspid regurgitation (TR) is standard, some neonates may have a trivial or even no TR jet despite presence of moderate to severe pulmonary hypertension [120]. To overcome the limitations of TR, studies utilize the Systolic Eccentricity Index (sEI) and the PAAT/RVET ratio (Pulmonary Artery Acceleration Time to Right Ventricular Ejection Time) to assess pulmonary pressure [29,121]. An sEI > 1.3 serves as a reliable objective measure of septal flattening and RV pressure overload [47]. Levy et al. (2016) reported that PAAT < 90 msec and PAAT/RVET <0.31 yielded high diagnostic accuracy for pulmonary hypertension (97% sensitivity and 95% specificity) [122]. Vasquez et al. showed that TH is associated with increased PVR, with an increase in sEI from 1.27 to 1.53 (*p* = 0.01) and PAAT:RVET ratio from 0.20 to 0.29 (*p* = 0.02) [121]. Additionally, Aggarwal and Natarajan showed that elevated diastolic eccentricity index in diastole was independently associated with mortality in their cohort, supporting other evidence that RV dysfunction and hemodynamic instability are associated with higher mortality and adverse outcomes [91].

#### 3.5.5. Impact of Cardiac Function on Clinical Outcome

Cardiac dysfunction, assessed by echocardiography, biomarkers, or need for cardiovascular support, has a significant impact on both short- and long-term outcomes in neonates with HIE (Table 6). Outcomes were classified into short-term outcomes (mortality prior to discharge, need for inotropic or extracorporeal support, and MRI-defined brain injury prior to discharge) and long-term outcomes (neurodevelopmental outcome at ≥12 months of age). RV dysfunction (e.g., reduced TAPSE and RV-FAC) and biventricular dysfunction independently predicted death or MRI brain injury [13,16,119]. LV GLS has emerged as a more sensitive echocardiographic predictor of brain injury, outperforming conventional indices [29]. Elevated cTnI at 24 h correlated with neurodevelopmental impairment at 18–22 months [61]. Each additional 24 h of inotropic support increased the odds of adverse short- and long-term outcomes by 14% and 12%, respectively [40,123]. The presence of persistent PH after birth was associated with higher mortality and greater brain injury burden [39]. Together, these findings underscore the prognostic value of early and comprehensive cardiovascular assessment in this population.

**Figure 4:**
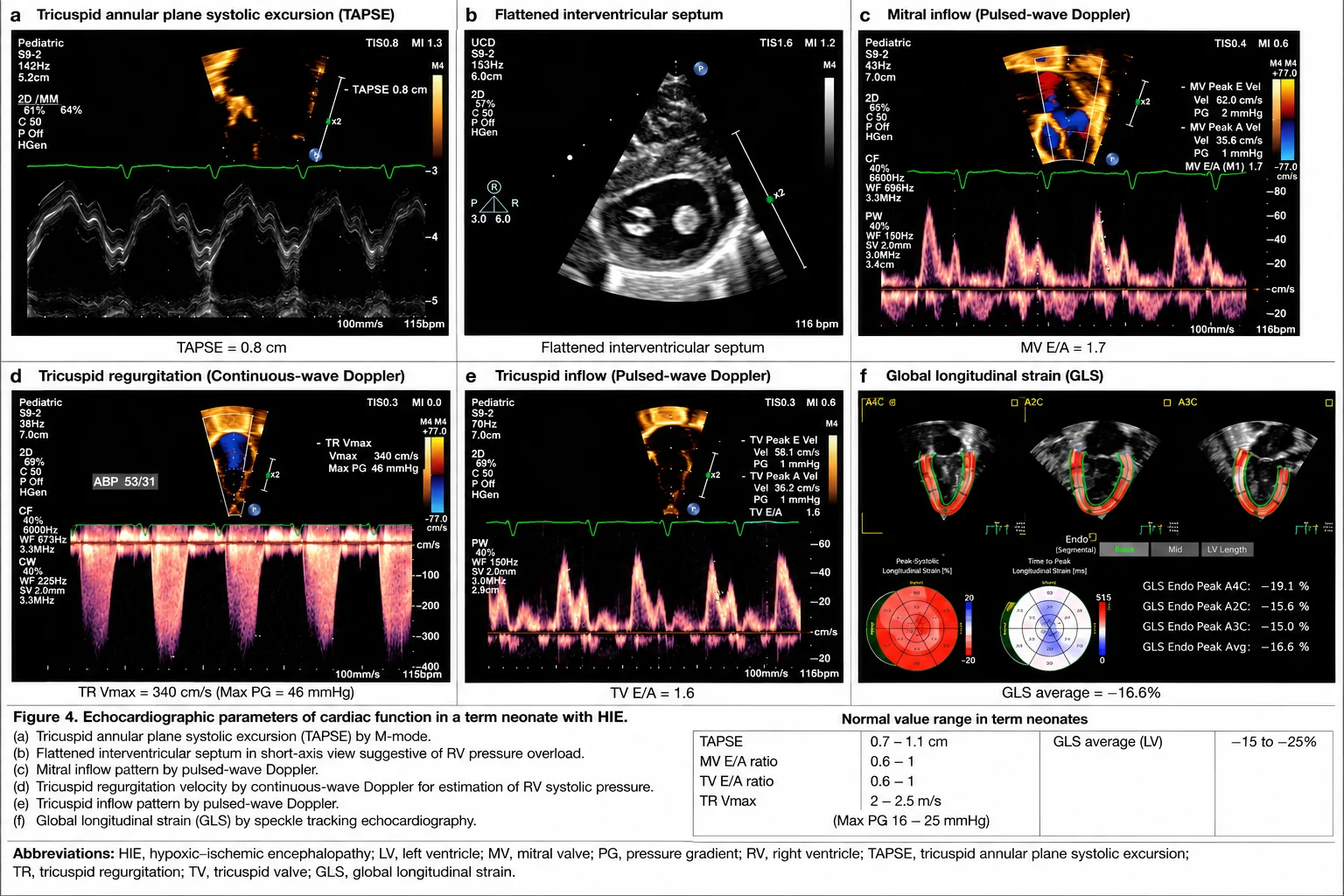
Echocardiographic assessment of cardiac function in a term neonate with hypoxic–ischemic encephalopathy.

Figure 4 illustrates several commonly used echocardiographic views and measurements employed to assess cardiac function in neonates with HIE. These parameters evaluate ventricular systolic and diastolic function, pulmonary hemodynamics, and myocardial deformation, providing a comprehensive assessment of cardiovascular status in this population.

## 4. Discussion

### 4.1. Overview

This narrative review synthesizes evidence from 81 studies on neonates with HIE and suggests that cardiovascular dysfunction is highly prevalent in infants with moderate to severe HIE and plays an important role in determining both short- and long-term outcomes. Cardiovascular involvement was identified in up to 70–90% of neonates when multi-modal diagnostic approaches were used, underscoring that myocardial injury is common and clinically relevant, warranting early recognition to guide timely management.

We acknowledge the wide variation across studies in both the reported prevalence of cardiac dysfunction among infants with HIE and the proposed biomarker thresholds. The prevalence of cardiac dysfunction ranges from approximately 29% to nearly 100%. This variation likely reflects differences in study populations, HIE severity, timing of assessment, sensitivity of diagnostic tests, and the criteria used to define cardiac dysfunction.

Infants with moderate-to-severe HIE are more likely to develop cardiac dysfunction than those with mild HIE. The timing of assessment, in relation to the hypoxic–ischemic insult, therapeutic hypothermia, and rewarming, may also affect the findings because cardiac function can improve during recovery. Differences in diagnostic methods may contribute further. Newer echocardiographic techniques, such as tissue Doppler imaging and speckle-tracking strain, are more sensitive for detecting cardiac dysfunction than conventional echocardiographic measures.

Similar factors likely explain the wide range of proposed troponin thresholds to define cardiac dysfunction. Troponin values may vary depending on the sensitivity of the assay and the timing of blood sampling, particularly because levels may peak approximately 12–24 h after the hypoxic–ischemic insult.

### 4.2. Clinical Markers (Blood Pressure, Heart Rate, Lactate, Urine Output)

A key finding of this review is the discordance between conventional clinical markers and true hemodynamic status. As previously stated, parameters such as blood pressure, heart rate, urine output, and lactate may not reliably reflect underlying myocardial function, particularly when interpreted in isolation. This limitation is particularly relevant during TH, where physiologic adaptations further obscure clinical assessment [19,24]. At times, lower blood pressure does not always indicate impaired perfusion. Over-reliance on mean arterial pressure may lead to inappropriate use of vasopressors such as dopamine, which can increase pulmonary vascular resistance and exacerbate RV afterload. Similarly, blood pressure may be maintained despite very poor cardiac output and risk of end-organ under perfusion. These findings highlight the importance of using multiple and better diagnostic markers to assess cardiovascular physiology. For each parameter, evidence assessment is provided based upon the authors’ judgment based on this narrative synthesis, not a formal GRADE or risk-of-bias appraisal.

Evidence assessment: Clinical markers alone are consistently reported as insensitive and are not supported as standalone diagnostic tools.

### 4.3. Electrocardiography and Cardiac Biomarkers

This review supports a shift toward a multimodal diagnostic approach integrating electrocardiography, cardiac biomarkers, and echocardiography. While electrocardiography remains a useful screening tool for arrhythmias and ischemic changes, its limited sensitivity and specificity, particularly in early or mild cardiac disease, restrict its standalone utility [47,48]. Cardiac biomarkers, particularly troponins which largely replaced CK-MB, provide sensitive detection of myocardial injury and correlate with disease severity and neurodevelopmental outcomes [12,31,127]. However, its clinical applicability is constrained by the lack of standardized thresholds. The reported cTnT cutoffs ranging from 0.1 to 0.25 µg/L [31,34,44,56,61,70]. In practice, biomarkers are best used in conjunction with imaging to guide further evaluation rather than as definitive diagnostic tools.

Evidence assessment: Electrocardiography is promising as an inexpensive adjunct (Jedeikin grading), especially in low-resource areas, but it is limited by weak sensitivity and specificity to be used as a standalone marker. Cardiac biomarkers (troponins) are supported as sensitive markers of injury severity across multiple independent cohorts, though threshold standardization remains uncertain.

### 4.4. Conventional and Advanced Echocardiography (Tissue Doppler, Speckle-Tracking Strain)

Echocardiography remains the cornerstone of cardiovascular assessment in infants with HIE. However, conventional indices of systolic function, such as EF and FS may lack sensitivity. In contrast, advanced echocardiographic techniques, including TDI and speckle-tracking strain, provide a more sensitive and physiologically relevant assessment. LV GLS consistently emerges as a strong predictor of adverse neurological outcomes, reflecting early subendocardial injury that is not captured by traditional metrics [47,108,117].

Evidence assessment: Conventional indices (EF, FS) show limited sensitivity, consistently reported across studies. Advanced techniques (TDI, speckle-tracking strain, particularly LV global longitudinal strain) are well supported but require expertise.

### 4.5. Right Ventricular Performance and Pulmonary Hypertension

The RV appears particularly vulnerable in this population as PVR fails to fall due to perinatal, even persistent, hypoxia and acidosis. It may be further elevated from therapeutic hypothermia, release of vasoactive mediators, or secondary to medications such as dopamine. This can lead to persistent pulmonary hypertension, causing a sustained increased afterload on the RV [17,18]. Pathological studies in asphyxiated neonates have documented focal myocardial necrosis, papillary muscle infarction, and subendocardial ischemia, that are most prominent in the RV and interventricular septum [128]. Conventional echocardiographic RV measures (e.g., TAPSE, FAC) and advanced strain parameters (e.g., RV FWS) consistently demonstrate RV dysfunction and are associated with adverse outcomes [16,47,96,118,119]. Several physiological factors help explain why RV performance, more so than LV performance, tends to carry the greatest prognostic weight in this population. In utero, the RV is the dominant ventricle and ejects most of the combined ventricular output against the high-resistance pulmonary circuit [129]. At birth, the normal transition requires the RV to rapidly adapt from a high-resistance PVR to lower pressures as PVR falls with lung aeration. This postnatal adaptation of the RV inherently lags behind that of the LV, making the RV uniquely vulnerable during the transitional period [130]. Its thin, crescent-shaped wall and its greater sensitivity to afterload, relative to more preload-dependent LV, make it disproportionately vulnerable to the sustained rise in PVR that characterizes HIE [16,131]. These findings suggest that RV dysfunction is not merely a marker of disease severity but may actively contribute to impaired pulmonary circulation and reduced cerebral oxygen delivery, subsequently affecting clinical outcomes in neonates with HIE.

Evidence assessment: RV dysfunction and elevated pulmonary pressures are consistently and independently associated with adverse outcomes across multiple cohorts, representing one of the more replicated findings in the literature.

### 4.6. Cardiac–Cerebral Interaction and Clinical Outcomes

Importantly, this review highlights an intricate interaction between the cardiovascular system and the brain in HIE. Myocardial dysfunction reduces cardiac output and potentially limits oxygen delivery to an already-injured brain, while neurological injury disrupts autonomic regulation of heart rate, vascular tone, and contractility [22,89,132,133]. This creates a self-reinforcing cycle in which a failing heart impairs cerebral perfusion, and an injured brain further destabilizes cardiovascular control. This framework provides a physiologic explanation for the consistent association between cardiac dysfunction and adverse neurological outcomes. Consistent with this concept, cardiac dysfunction, assessed by echocardiography, biomarkers, or need for cardiovascular support, is independently associated with mortality and neurodevelopmental impairment. RV dysfunction, biventricular dysfunction, elevated troponins, prolonged inotropic support, and persistent pulmonary hypertension are each associated with death or MRI-defined brain injury. The duration of cardiovascular support is particularly informative, with each additional 24 h of inotropic therapy associated with increased risk of adverse outcomes. These findings reinforce the importance of longitudinal cardiovascular assessment rather than reliance on single time point measurements. Recognizing the close relationship between cardiac function and cerebral perfusion highlights the importance of maintaining hemodynamic stability after asphyxia. Optimizing cardiovascular function may improve cerebral oxygen delivery and represents a potentially modifiable factor that could influence neurological outcomes after the initial insult.

Evidence assessment: The association between cardiac dysfunction and adverse neurological outcome is consistently reported.

### 4.7. Proposed Algorithm

**Figure 5:**
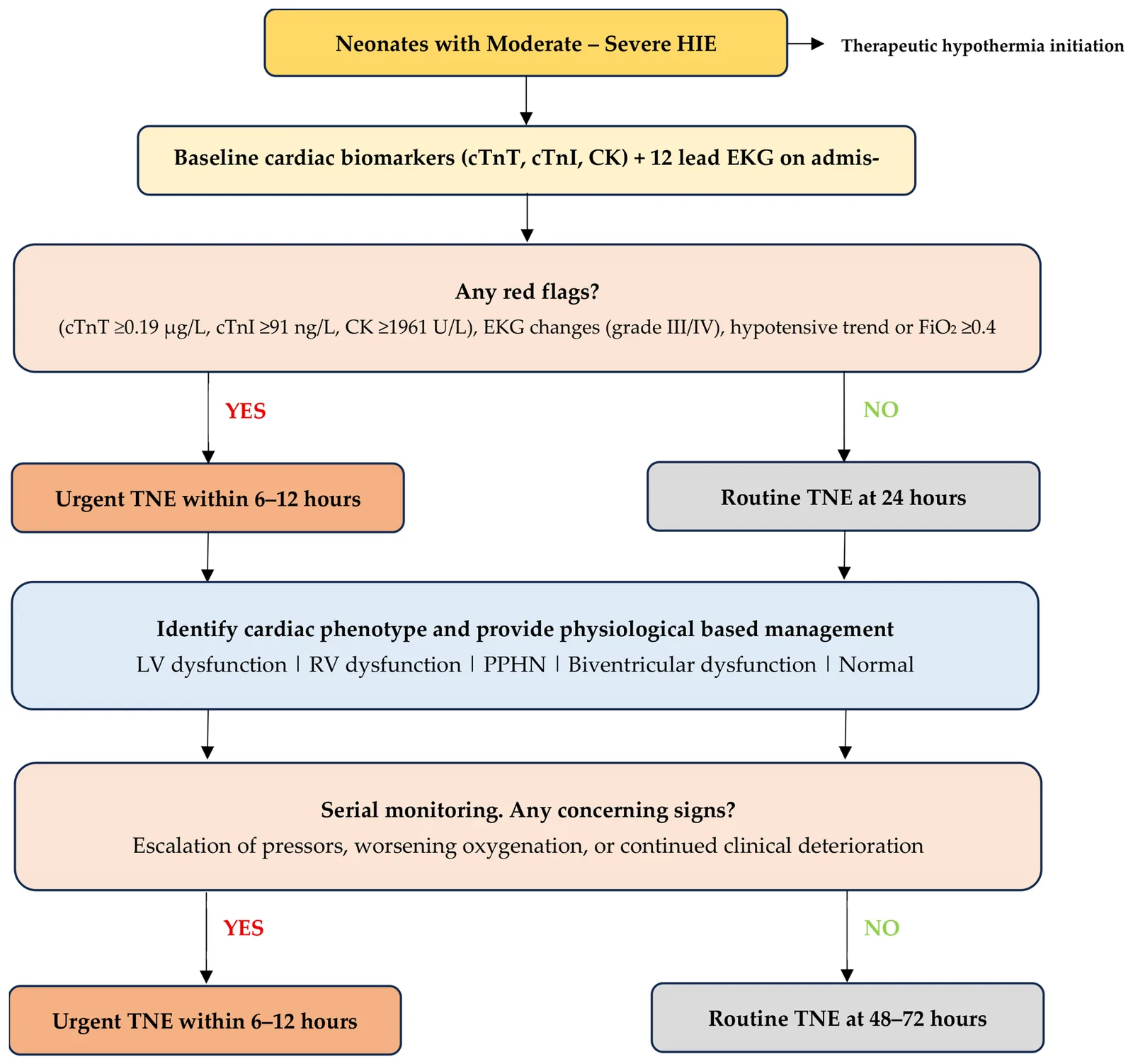
Proposed stepwise algorithm for the screening, diagnosis, and physiologically guided management of cardiac dysfunction in neonates with moderate-to-severe HIE undergoing therapeutic hypothermia. CK, creatine kinase; cTnI, cardiac troponin I; cTnT, cardiac troponin T; EKG, electrocardiogram; FiO_2_, fraction of inspired oxygen; HIE, hypoxic–ischemic encephalopathy; LV, left ventricle; PPHN, persistent pulmonary hypertension of the newborn; RV, right ventricle; TH, therapeutic hypothermia; TnECHO, targeted neonatal echocardiography.

Based on the evidence from this review, together with updated American Society of Echocardiography guidelines for targeted neonatal echocardiography (TnECHO) and cardiac point-of-care ultrasound (POCUS) [47], we propose a stepwise clinical algorithm for the identification and monitoring of cardiac dysfunction in infants with HIE (Figure 5).

All neonates with moderate-to-severe HIE should have cardiac biomarkers (cTnT, cTnI, CK) and a 12-lead EKG obtained on admission. Red-flag criteria, defined as cTnT ≥ 0.19 µg/L, cTnI ≥ 91 ng/L, or CK ≥ 1961 U/L (thresholds derived from Costa et al. 2007 [44] and Moore et al. 2025 [85], Jedeikin Grade III/IV EKG changes, a hypotensive trend, or FiO_2_ requirement ≥0.4, should trigger urgent targeted neonatal echocardiography (TnECHO) within 6–12 h of birth. In the absence of red flags, a standard TnECHO at 24 h is recommended. Echocardiographic findings are used to classify the predominant cardiac phenotype (LV dysfunction, RV dysfunction, PPHN, biventricular dysfunction, or normal), which then directs physiology-based management. Serial echocardiographic monitoring at 48–72 h during the cooling phase and at rewarming (72–96 h) is recommended given the known hemodynamic instability during these periods. Escalation triggers for urgent repeat echocardiography include worsening oxygenation, vasopressor escalation, or clinical deterioration.

We acknowledge that this algorithm is a clinically plausible framework derived from the narrative synthesis of observational evidence in this review, together with existing echocardiography guidelines; it has not itself been prospectively validated, and the specific thresholds proposed are drawn from individual studies rather than pooled or externally validated data. It should be regarded as a starting point for clinical discussion and local protocol development rather than a validated management pathway, pending prospective evaluation.

The diagnostic and management framework outlined in Figure 5 assumes availability of biomarker assays, a 12-lead EKG, and echocardiography performed by an experienced operator, resources that are unevenly distributed even within high-income countries and frequently unavailable in the low- and middle-income settings. Extending the applicability of this evidence to less-resourced units is therefore an important practical consideration.

Where echocardiography cannot be performed and only biochemical analysis is available, we would favor serial measurement of cTnT, cTnI, or total CK/CK-MB, since a rising trend over the first 24–48 h is more informative. A 12-lead EKG using the Jedeikin grading system is inexpensive, portable, and does not require imaging expertise, and Grade III/IV changes can be combined with the biomarker trend and standard clinical parameters to identify infants who need to be transferred to a center with echocardiography. For an infant with hypotension in a limited setting, we would suggest a stepwise approach: confirm a persistent trend rather than acting on a single reading; correct reversible contributors such as hypovolemia, hypoglycemia, or acid–base disturbance; and reserve inotropic therapy for infants whose hypotensive trend is accompanied by clinical evidence of poor perfusion, such as rising lactate levels or reduced urine output. As previously discussed, blood pressure alone correlates poorly with underlying cardiac function [23].

We recognize the inconsistencies with clinical exam and indirect markers such as lactate levels and urine output in diagnosing cardiac dysfunction in neonates with HIE, but when echocardiography is not available, this biomarker- and EKG-based approach, together with close clinical observation and early discussion with a referral center, is the most pragmatic alternative.

When basic echocardiography is available, but the operator is not trained in strain imaging or advanced hemodynamic assessment, evaluation can focus on a few clinically useful qualitative findings. These include visual assessment of ventricular contractility, interventricular septal position as an indirect marker of elevated pulmonary vascular resistance, and the direction of shunting across the ductus arteriosus or foramen ovale. Together, these findings may help identify cardiac dysfunction and pulmonary hypertension. Serial qualitative assessments, interpreted alongside trends in cardiac biomarkers, may further improve diagnostic accuracy.

## 5. Limitations

This narrative review has several limitations that should inform interpretation of its findings. No prospective review protocol was registered. No formal quality or risk-of-bias appraisal tool (e.g., the Newcastle–Ottawa Scale) was applied to individual studies, and study selection and the relative weight given to individual studies in the narrative synthesis reflect the authors’ thematic judgment rather than a pre-specified, reproducible weighting scheme.

The included literature is characterized by substantial heterogeneity in the definition of cardiac dysfunction, diagnostic thresholds, and timing of assessment relative to the hypoxic–ischemic insult and TH; this heterogeneity, further compounded by variable and inconsistently reported short- and long-term outcomes, precluded a quantitative meta-analysis. Most included primary studies are single-center, observational, and retrospective, with comparatively small sample sizes (minimum 20 neonates per study by our eligibility criteria); as such, the findings summarized in this review should be interpreted as associations rather than as evidence of prediction or causality, unless a specific study’s design supports stronger inference. Long-term neurodevelopmental outcomes are inconsistently reported across studies, and data from low- and middle-income countries remain underrepresented despite the high global burden of HIE in these settings.

Because a subset of included studies represents secondary analyses of overlapping patient cohorts (for example, companion publications from the same research group reporting different cardiac endpoints from a shared population), we have not reported an aggregate neonate sample size across the 81 included studies.

Finally, while the proposed stepwise algorithm (Figure 5) integrates thresholds and diagnostic patterns reported across the included literature, these thresholds have not been prospectively validated as a unified decision tool. We reiterate that the algorithm should be interpreted as a clinically plausible, evidence-informed framework rather than a validated management pathway, pending future prospective study.

## 6. Conclusions

This review highlights the importance of early detection of cardiovascular dysfunction and how it influences the clinical trajectory of neonates with moderate to severe HIE. Despite its impact on mortality and co-morbidities, the early and reliable identification of cardiac dysfunction remains challenging due to the reliance on insensitive clinical markers. Such diagnostic unreliability underscores the necessity to shift toward multimodal monitoring, in which TnECHO and high-specificity cardiac biomarkers may serve as central diagnostic modalities. Future research should focus on prospective, multicenter studies with standardized definitions and protocols, including validated biomarker thresholds and uniform echocardiographic assessment. The integration of advanced imaging techniques into routine clinical workflows warrants further evaluation and research of their impact, especially on the long-term outcomes. Critically, prospective multi-center large interventional studies are needed to determine whether physiology-guided cardiovascular management improves neurological outcomes.

## Figures and Tables

**Table 1:** The Jedeikin grading system for electrocardiographic classification of myocardial ischemia in neonates, based on T-wave morphology, ST-segment deviation, and Q-wave abnormalities across four severity grades [42].

**Grade I—Flat/Inverted T wave in 1–2 Leads**	
T wave	Flat or inverted in 1–2 leads (aVR excluded)
ST segment	Normal
Q wave	Normal
**Grade II—Flat/Inverted T wave in > 3 Leads**	
T wave	Flat or inverted in > 3 leads (aVR excluded)
ST segment	Normal
Q wave	Normal
**Grade III—T-wave changes + ST segment changes or Q wave changes**	
T wave	Flat or inverted in > 3 leads (aVR excluded)
ST segment	Depression or elevation >2 mm in ≥2 chest leads, OR >1 mm in ≥2 standard leads
Q wave	Duration > 0.02 s OR amplitude >25% of R wave, in ≥1 anterior or ≥3 related chest leads
**Grade IV—Either pattern**	
Pattern A	Pathological Q waves AND markedly elevated ST segment
Pattern B	Complete left bundle branch block (LBBB)

**Table 2:** Summary of published studies evaluating electrocardiographic changes in neonates with HIE, including sample sizes, EKG parameters assessed, key conclusions, and methodological limitations.

Author (Year)	Sample Size (n)	EKG Parameters Assessed	Conclusion	Limitations
Jedeikin et al. (1983) [42]	61	• T-wave axis and amplitude • ST segment changes • Q-wave abnormalities (Grade 3–4)	• Developed the Jedeikin 4-grade classification of myocardial ischemia • T-wave assessment unreliable before 24 h • Sensitivity of the EKG as a detector of myocardial ischemic damage at 5–8 h is only 40% and the specificity is 81%	• T-wave assessment unreliable before 24 h • ‘Stressed’ defined clinically; no formal HIE grading • Grade 1 changes seen in some healthy neonates; low specificity at mild threshold
Tapia-Rombo et al. (2000) [49]	40	• Jedeikin criteria (Grade 1 excluded; >=Grade 2 required for diagnosis) • T-wave flat/inverted; ST depression/elevation; Q-wave abnormality • Conduction abnormalities: RBBB, LBBB, hemiblocks, AV block • Changes required persistence >= 48 h • TMI (Transient myocardial Ischemia) diagnosis required: EKG changes + elevated CPK-MB	• TMI is more common than previously reported (82.5%). • EKG should be performed in all asphyxiated neonates alongside CPK-MB measurement	• No formal Sarnat HIE staging • CPK-MB non-specific in neonates
Boo et al. (2005) [31]	100	• Jedeikin 4-grade classification on days 1 and 2 of life • T-wave flat/inverted; ST depression/elevation; Q-wave abnormality • EKG correlated with serial cTnT and CK-MB	• EKG abnormality is not discriminatory for cardiac dysfunction severity, heart failure, low EF, or mortality in asphyxiated neonates. • cTnT superior to EKG and CK-MB for detecting clinically significant cardiac dysfunction	• Reference range for normal cTnT varied across published studies
Costa S et al. (2007) [44]	59	• Jedeikin 4-grade classification on day 1 of life • T-wave flat/inverted; ST depression/elevation; Q-wave abnormality • EKG correlated simultaneously with echocardiographic findings and cTnT	• EKG was insensitive for detecting myocardial ischemia in asphyxiated neonates, producing only non-specific grade 1–2 changes even when echo confirmed myocardial insult in 79% of cases	• Includes preterm infants • EKG assessment limited to first 24 h
Kanik et al. (2009) [45]	34	• Jedeikin 4-grade classification on days 1–2 of life • EKG changes correlated with LV ejection fraction (echo, EF <60% = abnormal); CK-MB; cTnI on days 1 and 3	• Abnormal EKG significantly predicts neonatal mortality in HIE (Sens 77%, Spec 76%, PPV 53%, NPV 90%) • EKG changes correlated with advanced HIE stage and multiorgan involvement	• Small sample (n = 34 HIE neonates) • Only LVEF assessed by echo; no RV function evaluation • No long-term neurodevelopmental follow-up
Rajakumar et al. (2009) [50]	60	• ST segment depression, T wave inversion, T wave flattening, and the presence of significant Q waves	• Both EKG and echocardiography are useful tools for evaluating the severity of myocardial damage in newborns with perinatal asphyxia	• The study lacked objective assessments of ventricular dysfunction through measurement of Right and Left systolic Time Intervals (RVSTI and LVSTI) to precisely define hypokinesia • There was a possibility that the use of medications (such as high-dose phenobarbitone for seizures) might have contributed to the observed cardiac dysfunction • The selection of more severely asphyxiated babies may have led to a higher reported incidence of myocardial dysfunction compared to other literature
Agrawal et al. (2012) [30]	60	• Jedeikin 4-grade classification within 72 h of life • EKG correlated with CK-Total, CK-MB, Troponin I	• EKG changes common (76.7%), correlate with HIE severity, and predict poor outcome. • Combined EKG + enzyme assessment improves diagnostic yield	• No control (non-asphyxiated) group • No echocardiographic correlation • Single center study
Bhasin & Kohli (2019) [28]	23	• Jedeikin 4-grade classification within 24–48 h of life • EKG correlated with CK-T, CK-MB, troponin-T, Echo	• All severely asphyxiated neonates had EKG changes; EKG was more sensitive than echo.	• Only severely asphyxiated neonates enrolled; limits generalizability • Qualitative rather than quantitative troponin-T assay used
Yellanthoor and Dineshkumar (2021) [51]	57	• T wave morphology, ST segment changes, Q-wave abnormalities, Conduction issues	• EKG changes occurred in 77.1% of asphyxiated neonates, while nearly half (45.6%) showed echocardiographic changes • Routine EKG and echocardiographic evaluations are valuable for understanding the extent of cardiac involvement and aiding in prognostication and parental counseling	• Certain severely asphyxiated neonates were excluded from the analysis because they were on high-frequency oscillatory ventilation (HFOV), which prevented the team from obtaining EKG and echocardiography data

HIE, hypoxic–ischemic encephalopathy; CK-MB, creatine kinase-MB; cTnI, cardiac troponin I; cTnT, cardiac troponin T; EF, ejection fraction; NPV, negative predictive value; PPV, positive predictive value; TMI, transitory myocardial ischemia.

**Table 3:** Key studies evaluating cardiac biomarkers in neonates with HIE, including sample sizes, biomarkers assessed, diagnostic thresholds applied, and principal conclusions. Studies are stratified by biomarker type: cardiac troponin T (cTnT), cardiac troponin I (cTnI), and creatine kinase-related markers. Threshold values are reported as published. Absence of a uniform threshold across studies reflects the key methodological limitation identified in this review.

Author	Sample Size (n)	Biomarker Assessed	Threshold Level	Conclusion
Boo NY et al. (2005) [31]	100	cTnT, CK-MB	cTnT > 0.1 µg/L	• cTnT is significantly higher in asphyxiated infants who die or develop cardiac dysfunction • CK-MB is not discriminatory • cTnT > 0.1 µg/L is the threshold for identifying heart failure with 72.7% sensitivity, 36% specificity and for detecting low ejection fraction with sensitivity 89% and specificity 39%
Szymankiewicz et al. (2005) [52]	83	cTnT	cTnT > 0.1 µg/L	• While asphyxiated infants had significantly higher cTnT levels and more frequent tricuspid insufficiency, other echocardiographic parameters like cardiac output and fractional shortening remained in the same range as healthy infants • Infants requiring inotropic support had significantly higher cTnT levels (0.397 ± 0.206 vs. 0.091 ± 0.135, *p* < 0.05)
Costa S et al. (2007) [44]	59	cTnT	cTnT > 0.19 µg/L	• cTnT is elevated in asphyxiated neonates and correlates with echocardiographic myocardial damage • cTnT cut-off value of 0.19 µg/L demonstrated 100% specificity and 57% sensitivity for echocardiographic abnormalities. However, the authors noted that this threshold was not consistently associated with clinical myocardial dysfunction or cardio-circulatory compromise, as prematurity itself may significantly contribute to elevated cTnT levels
Uzodimma et al. (2012) [53]	60	cTnT	cTnT > 0.1 µg/L	• Serum cTnT showed strong negative correlations with umbilical arterial pH (r = −0.75), 5 min Apgar score (r = −0.74), and 1 min Apgar score (r = −0.70), indicating that more severe asphyxia was associated with greater myocardial injury as reflected by higher cTnT
Sadoh et al. (2014) [54]	80	cTnT, CK-MB	cTnT > 0.012 µg/L	• Renal and myocardial injuries frequently co-exist in cases of severe perinatal asphyxia. cTnT was significantly elevated in myocardial injury but not significantly elevated in AKI alone • CK-MB was significantly elevated in AKI (*p* < 0.0001) and in combined injuries, but notably was not significantly different between infants with and without myocardial injury alone
Yildirim et al. (2016) [55]	60	cTnT, CK-MB	Not specified	• Troponin T is a useful, simple, and sensitive test for detecting cardiac damage in hypoxic neonates
Abiramalatha et al. (2017) [56]	63	cTnT	cTnT > 0.19 µg/L	• cTnT concentration on day 1 of life has a good predictive value for mortality before discharge. • cTnT cut-off value of 0.19 µg/L had 82% sensitivity and 80% specificity for predicting mortality before discharge
Joseph S et al. (2018) [57]	120	cTnT	• cTnT > 0.1 µg/L—cardiac dysfunction • cTnT > 0.2505 µg/L—Mortality	• cTnT measured at 4 h of life is a highly accurate early biomarker for myocardial dysfunction, shock, severe HIE, and mortality in asphyxiated neonates • cTnT > 0.2505 µg/L predicts mortality with 84% sensitivity and 96.6% specificity
Sweetman et al. (2020) [58]	54	cTnT	• cTnT thresholds: • Day 1: 0.84 µg/L • Day 2: 0.63 µg/L • Day 3: 0.58 µg/L	• Elevated levels are highly significant predictors of mortality and correlate with the severity of encephalopathy, occurrence of seizures (day 1 and day 2), and brain injury on MRI (day 3) • Serial measurements up to day 3 of life are more useful than a single day 1 measurement for identifying infants who require close cardiovascular monitoring
Yellanthoor RB & Rajamanickam D (2022) [34]	57	cTnT	cTnT > 0.15 µg/L	• cTnT > 0.21 µg/L is significantly associated with echo abnormalities, and inotrope requirement • Higher cTnT levels associated with HIE severity, and non-survival • cTnT is elevated in 58% of neonates with normal echo, supporting its use even in the absence of echocardiographic changes
Ünsal et al. (2023) [59]	111	cTnT, CK, CK-MB	cTnT > 164 ng/L	• A cut-off value of 164 ng/L for Troponin-T was determined to predict mortality (77% sensitivity, 67% specificity) • cTnT serves as a valuable prognostic marker for mortality and clinical outcomes
Abdelrahman et al. (2025) [60]	48	cTnT	• cTnT > 0.18 ng/mL—detecting myocardial dysfunction and mortality • cTnT > 0.15 ng/mL—identifying need for inotropic support	• Elevated cTnT is highly suggestive of myocardial dysfunction in HIE • It demonstrates a significant correlation with the severity of HIE stages, morbidity, and mortality in asphyxiated neonates
Liu X et al. (2013) [61]	75	cTnI	• cTnI < 0.15 µg/L—Hypothermia • cTnI < 0.22 µg/L—Normothermia (associated with good outcomes)	• cTnI at threshold levels at 24 h of age is a valid prognostic marker for neurodevelopmental outcome at 18–22 months in both normothermic and hypothermic infants • TH is cardioprotective, significantly reducing cTnI release
Trevisanuto et al. (2006) [62]	52	cTnI	• cTnI > 0.15 µg/L	• cTnI levels do not correlate with traditional markers of asphyxia such as umbilical pH, Apgar scores, or QTc interval • The authors offer two possible explanations for the lack of correlation: (a) the small sample size (n = 13 asphyxiated neonates), or (b) the intrinsic heterogeneity of perinatal asphyxia as a spectrum of conditions with diverse organ involvement, severity, duration, and chronicity
Türker et al. (2004) [63]	104	cTnI, CK, CK-MB	Cord blood cTnI > 4.6 µg/L	• Significant elevation of cord blood cTnI is an excellent early predictor of both the severity of HIE and mortality in term infants • CK and CK-MB did not differentiate between no/mild HIE and moderate/severe HIE subgroups at most time points
Kanik E et al. (2009) [45]	34	cTnI, CK-MB	Not specified	• Day-3 cTnI is significantly higher in non-survivors, but sensitivity for mortality prediction is limited (33%) • EKG changes and cTnI level at 72 h after birth are likely to have significant predictive value in the assessment of mortality in HIE • CK-MB is not a useful predictor of mortality in HIE
Shastri et al. (2012) [64]	60	cTnI	• Grade 1 HIE: 0.04 μg/L • Grade 2 HIE: 0.12 μg/L • Grade 3 HIE: 0.67 μg/L • (median concentration)	• cTnI concentrations measured within the first 36 h of birth correlate strongly with the clinical grade of HIE and the duration of inotropic support required • early cTnI levels provide a useful proxy marker for anticipating the severity of myocardial dysfunction in asphyxiated neonates
Montaldo et al. (2014) [65]	178	cTnI, CK-MB	• cTnI > 0.58 µg/L—Adverse neurodevelopmental outcome • cTnI > 8.1 µg/L—Mortality	• Early cTnI concentration (within 12 h of birth) correlates with severity of HIE, myocardial dysfunction and with Bayley II scores at 18 months • CK-MB was not a useful predictor • No significant difference in cTnI concentrations was found between cooled and non-cooled infants (*p* > 0.05)
Mangshetty et al. (2014) [66]	100	cTnI	• cTnI < 14 pg/L—Normal	• Early cTnI concentrations (within 72 h of birth) correlate strongly with the clinical grade of HIE (Mean levels—Stage I (9.56 ± 5.05 pg/L), Stage II (49.84 ± 15.88 pg/L), and Stage III (106.9 ± 31.01 pg/L) • cTnI is a useful marker for predicting the severity of myocardial damage and can help predict the outcome (mortality) in asphyxiated neonates
Simovic et al. (2014) [67]	91	cTnI, total CK and CK-MB	• cTnI > 0.135 µg/L	• cTn I was the only biochemical marker that significantly differentiated non-survivors from survivors • Total CK, CK-MB, and CRP did not show significant differences between the two asphyxia subgroups
Zhou et al. (2017) [68]	120	cTnI, CK-MB, NT-proBNP	• cTnI > 0.1 µg/L	• Serum cTnI levels measured within the first hours of life are significantly elevated in asphyxiated neonates compared to controls and correlate with the severity of asphyxia • cTnI was the strongest predictor of mortality among the biomarkers tested, outperforming CK-MB
Jiang et al. (2019) [69]	59	hs-cTnI, CK-MB	• hs-cTnI > 0.087 µg/L at 12 h of life	• Serum hs-cTnI levels can predict myocardial injury caused by neonatal asphyxia at an early stage • Despite the low sensitivity of hs-cTnI, it showed a considerably higher specificity of 95.5% and diagnostic accuracy of 77.5%
Lee et al. (2021) [70]	73	cTnI, CK, CK-MB	• cTnI ≥ 0.06 µg/L • cTnI ≥ 0.12 µg/L • cTnI ≥ 0.18 µg/L	• cTnI cut-off level of 0.06 µg/L predicted moderate to severe HIE with a sensitivity of 77% and specificity of 68% • cTnI cut-off level of 0.12 µg/L predicted moderate-to-severe HIE with higher PPV (90.9%) and specificity (90.5%) than the 0.06 µg/L cutoff, and also predicted hypotension • cTnI cut-off level of 0.18 µg/L was strongly associated with hypotension during first admission and significantly related with abnormal neurodevelopmental outcomes at 1 year
Issa et al. (2021) [71]	170	cTnI	• cTnI ≥ 1.26 µg/L • cTnI (median) 3.58 µg/L—HIE stage III • cTnI (median) 4 µg/L—non survivors	• Cardiac troponin I is significantly higher in asphyxiated subjects compared with healthy controls • Elevated levels are strongly associated with higher mortality and can help serve as a prognostic indicator of the severity of HIE
Munshi et al. (2022) [72]	104	cTnI	• cTnI ≥ 0.12 µg/L	• A cut-off of cTnI ≥ 0.12 µg/L was identified as the optimal threshold for predicting any residual encephalopathy at discharge, with a sensitivity of 77%, specificity of 78%, positive predictive value of 68%, and negative predictive value of 84% • Early elevation of cTnI (within 6 h of age) predicts a high risk of having residual encephalopathy at discharge
Xu et al. (2022) [73]	201	cTnI, procalcitonin (PCT), CK-MB	• cTnI > 0.102 µg/L • CK-MB > 30.655 U/L • PCT > 3.223 µg/L	• cTnI demonstrated the highest predictive ability for diagnosing HIE post-perinatal asphyxia compared to PCT, CK-MB, and lactate
Nwankwo O et al. (2024) [74]	120	cTnI	Not specified	• cTnI levels are significantly elevated in asphyxiated neonates with HIE and correlate positively with HIE severity
Sero et al. (2025) [75]	115	cTnI, CK-MB, NT-proBNP	• cTnI ≥ 0.428 µg/L	• A cTnI threshold of 0.428 ng/mL was identified for predicting mortality, with 87.5% sensitivity and 87.2% specificity • cTnI levels analyzed in the first hours of life in newborns with perinatal asphyxia have predictive value for both mortality and brain injury on imaging
Marzban et al. (2025) [76]	84	cTnI	• cTnI ≥ 14 pg/mL	• cTnI levels were significantly higher in infants who required advanced resuscitation compared to those who only received early resuscitation and positive pressure ventilation • Elevated cTnI levels also correlated with lower Apgar scores at 5 and 10 min, lower pH, and worse base excess
Pabra et al. (2026) [77]	100	cTnI	• cTnI > 0.3 µg/L	• cTnI levels within the first 6 h of life serve as a sensitive and early biochemical marker for ischemic myocardial injury • Elevated levels show a statistically significant correlation with morbidity and mortality, particularly in infants experiencing shock and requiring inotropic support
Elobuike et al. (2026) [78]	138	cTnI, cTnT	• cTnI ≥ 1.6 ± 0.2 µg/L • cTnT ≥ 92.7 ± 47.9 µg/L	• Mean levels of both cTnT and cTnI are significantly higher in asphyxiated neonates than in normal controls • While both markers are elevated, cTnI demonstrated a significant negative correlation with Apgar scores, indicating it may be more sensitive to the degree of asphyxia than cTnT
Gonchar et al. (2020) [79]	94	cTnI, copeptin, ischaemia-modified albumin (IMA)	• IMA > 2279.96 µg/L (general asphyxia) > 2386.87 µg/L (severe asphyxia)	• Copeptin and IMA levels in umbilical cord blood could provide an opportunity for early prediction of post-hypoxic myocardial damage and cardiovascular disorders in newborns • Copeptin had notably high sensitivity (87.2%) but low specificity (41.7%) for general asphyxia, while for severe asphyxia it had high specificity (97.7%) but low sensitivity (50.0%)
Agrawal J et al. (2012) [30]	60	CK Total, CK-MB, cTnI	• CK Total > 190 U/L • CK-MB > 25 U/L • cTnI > 0.05 µg/L	• CK-MB and TnI are elevated in the majority of HIE neonates and correlate with HIE severity and mortality • CK Total was elevated in 90% of neonates and correlated with HIE severity (*p* = 0.02), but notably did not significantly correlate with mortality (*p* = 0.674)
Bhasin H & Kohli C (2019) [28]	23	CK Total, CK-MB, cTnT	• CK Total > 190 U/L • CK-MB > 25 U/L • cTnT > 0.1 µg/L	• CK Total and CK-MB were raised in all 23 cases (100%), and mean serum levels of CK-T, CK-MB (*p* < 0.001), and cTnT (*p* = 0.002) were higher in non-survivors
Kumar et al. (2023) [80]	70	CK-MB fraction	• Birth—13.14 ± 12.03 U/L • 24 h—40.96 ± 25.92 U/L • 72 h—27.97 ± 14.87 U/L • (mean values)	• 83.3% of asphyxiated babies with shock had elevated CK-MB levels at birth (*p*-value < 0.001), but CK-MB values were not significantly elevated at 24 and 72 h
Yang et al. (2023) [81]	90	CK, and CK-MB, cTnT, cTnI)	• CK-MB > 135.4 U/L • cTnT > 112.6 ng/L • cTnI > 55.3 ng/L	• Cord blood levels of CK-MB, cTnT, and cTnI are significantly elevated in asphyxiated neonates and demonstrate significant discriminatory ability for the early, non-invasive prediction of myocardial injury
Gupta et al. (2024) [82]	240	CK-MB	• CK-MB > 92.6 U/L at 8 h of life • CK-MB > 60 U/L at 24 h of life	• CK-MB elevation and reduced systolic function (assessed by echocardiography) are considered the most reliable markers of ischemia • Raised CK-MB levels were significantly associated with both the severity of HIE and abnormal echocardiographic findings
Kumavat et al. (2024) [83]	100	CK-MB, LDH	• CK-MB at 8 h: >92.6 U/L • CK-MB at 24 h: >60 U/L • LDH (at 72 h): >580 U/L	• CK-MB and LDH levels are significantly elevated in asphyxiated neonates and show a statistically significant correlation with the severity HIE • CK-MB at 8 h: sensitivity 76%, specificity 83%, PPV 92%, NPV 100% • LDH at 72 h: sensitivity 83%, specificity 96%, PPV 89%, NPV 96% • ROC AUC values: CK-MB at 8 h = 0.921, LDH at 72 h = 0.954
Karuthapandy et al. (2025) [84]	135	CK-MB, CK total, cTnI	No specific threshold levels	• There is a significant positive correlation between the levels of all three cardiac markers (CK-MB, CK total, and cTnI) and the severity of HIE staging
Moore SS et al. (2025) [85]	56	CK Total, cTnI, Urotensin-II	• CK > 1961 U/L • cTnI > 0.091 µg/L	• Elevated levels (beyond threshold) of CK and cTnI are associated with decreased RV performance • High NPV of both markers (98% for CK; 96% for cTnI at TAPSE threshold) makes them useful for ruling out severe RV dysfunction • Urotensin-II showed no functional correlation
Hu X.L et al. (2025) [86]	53	CK-MB, cTnT, Myoglobin (Myo), cTnI, NT-proBNP	Not specified	• Standard serum biomarkers (CK-MB, cTnT, Myo, cTnI, NT-proBNP) did NOT significantly differentiate mild from severe asphyxia

CK, creatine kinase; CK-MB, creatine kinase-MB isoenzyme; HIE, hypoxic–ischemic encephalopathy; LDH, lactate dehydrogenase; NDI, neurodevelopmental impairment; NPV, negative predictive value; NT-proBNP, N-terminal pro-B-type natriuretic peptide; TAPSE, tricuspid annular plane systolic excursion; TH, therapeutic hypothermia.

**Table 4:** Echocardiographic parameters used across included studies to assess cardiac function in neonates with HIE, organized by left ventricular systolic and diastolic function, right ventricular systolic function, pulmonary hemodynamics, and advanced imaging modalities.

**Conventional Echocardiography Parameters**	
**Left Ventricular (LV) Systolic Function**	
Ejection Fraction (EF)	Calculated via Simpson’s biplane method or M-mode
Fractional Shortening (FS) or Shortening Fraction (SF)	Global markers of radial contractility
LV Output (LVO) and Stroke Volume (SV)	Determined by measuring the aortic valve annulus and velocity time integral (VTI)
Mitral Annular Plane Systolic Excursion (MAPSE)	Longitudinal displacement of the mitral annulus
Dimensions	Left ventricular internal diameters at end-diastole (LVIDd) and end-systole (LVIDs); end-diastolic and end-systolic volumes (EDV/ESV)
**Right Ventricular (RV) Systolic Function**	
Tricuspid Annular Plane Systolic Excursion (TAPSE)	Longitudinal movement of the tricuspid annulus toward the cardiac apex during systole
Fractional Area Change (FAC)	Global RV assessment involving manual tracing of the RV area in diastole and systole
RV Output (RVO) and Stroke Volume (SVRV)	Derived from the pulmonary valve annulus and RV outflow tract VTI
**Diastolic Function (Conventional Doppler)**	
Mitral and Tricuspid Inflow (E/A ratio)	Early-to-atrial filling velocity ratios
Isovolumetric Relaxation Time (IVRT) and Contraction Time (IVCT)	Durations measured from flow signals
**Pulmonary Hemodynamics**	
Estimated Systolic Pulmonary Arterial Pressure (sPAP)	Derived from tricuspid regurgitation (TR) jet velocity or patent ductus arteriosus (PDA) systolic velocity gradient
Eccentricity Index (EI or sEI)	Ratio of perpendicular LV diameters distal to the mitral valve to quantify septal flattening or bowing
Pulmonary Artery Acceleration Time (PAAT) and PAAT/RVET Ratio	Indicators of pulmonary vascular resistance
Shunt Characteristics	Direction, size, and velocity of flow across the PDA and Foramen Ovale (PFO)
**Advanced Echocardiographic Techniques**	
**Tissue Doppler Imaging (TDI)**	
Myocardial Velocities	Peak systolic (s′), early diastolic (e′), and late diastolic (a′) velocities of the LV lateral wall, RV free wall, or septum
Ratios	E/e′ ratio (marker of filling pressure) and E′/A′ ratio
Myocardial Performance Index (MPI or Tei index)	A combined indicator of systolic and diastolic performance (calculated as [IVCT + IVRT]/Ejection Time)
Time Intervals	TDI-derived IVRT′, IVCT′, ejection time (ET′), and filling time (FT′)
**Speckle-Tracking Echocardiography (STE)**	
Strain Parameters	Peak Global Longitudinal Strain (GLS), Peak Longitudinal Strain (pLS), and Right Ventricular Free Wall Strain (RV FWS)
Strain Rate (SR)	Global and segmental systolic, early diastolic, and atrial SR
Rotational Mechanics	Basal and apical rotation, net twist, torsion (twist/length), and untwist rate
Myocardial Work (MW)	Global Work Index (GWI), Global Constructive Work (GCW), Global Wasted Work (GWW), and Global Work Efficiency (GWE)
**3D Echocardiography**	
Evaluates LV end-diastolic volume (EDV), stroke volume (SV), ejection fraction (EF), muscle mass, and systolic desynchrony index with greater precision than 2D methods	
**Cerebral and Systemic Flow Adjuncts**	
Superior Vena Cava (SVC) Flow	A surrogate marker for systemic blood flow to the upper body and brain
Descending Aorta Blood Flow (DABF) and Resistive Index (RI)	Markers of lower body perfusion and peripheral vascular resistance
Cerebral Doppler	Assessment of the Middle Cerebral Artery (MCA) for peak systolic velocity (PSV), end-diastolic velocity (EDV), and resistive index (RI) to detect “luxury perfusion” or loss of autoregulation

DABF, descending aorta blood flow; EDV, end-diastolic volume; ESV, end-systolic volume; ET, ejection time; FAC, fractional area change; FT, filling time; GLS, global longitudinal strain; GWE, global work efficiency; GWI, global work index; IVCT, isovolumic contraction time; IVRT, isovolumic relaxation time; LV, left ventricle; LVIDd, left ventricular internal diameter at end-diastole; LVIDs, left ventricular internal diameter at end-systole; LVO, left ventricular output; MAPSE, mitral annular plane systolic excursion; MCA, middle cerebral artery; MPI, myocardial performance index; PAAT, pulmonary artery acceleration time; PDA, patent ductus arteriosus; PFO, patent foramen ovale; RI, resistive index; RV, right ventricle; RVET, right ventricular ejection time; RVO, right ventricular output; sEI, systolic eccentricity index; sPAP, systolic pulmonary arterial pressure; SR, strain rate; STE, speckle-tracking echocardiography; SV, stroke volume; SVC, superior vena cava; TAPSE, tricuspid annular plane systolic excursion; TDI, tissue Doppler imaging; VTI, velocity time integral.

**Table 5:** Key echocardiographic studies in neonates with HIE, organized by imaging modality and era, reporting sample sizes, parameters assessed, key findings, and principal conclusions.

Study	Sample Size (n)	Key Parameters	Key Findings	Conclusion
**Conventional Echocardiography (2D echo + Doppler): Pre-therapeutic Hypothermia Era**				
Tapia-Rombo et al. (2000) [49]	40	EF, diastolic/systolic dysfunction, tricuspid valve insufficiency; EKG for ischemia classification	• Transitory Myocardial Ischemia (TMI) diagnosed in 33/40 (82.5%) • The average myocardial ejection fraction was 74% ± 7% (range 60–90%) • Mortality in the TMI group was 24.2%, and the presence of EKG changes (subendocardial/subepicardial pattern) was significantly higher in neonates who died	• TMI is far more common than historically reported and often clinically silent • Asphyxiated neonates should be submitted to serial EKGs, total CPK, and CPK-MB fraction measurements, complemented by clinical findings and chest X-rays, to ensure early detection and opportune treatment
Kanik et al. (2009) [45]	34	LVEF (<60% = abnormal); comparison with EKG grades and biomarkers for mortality prediction	• Echo EF abnormal in only 1/34 (2.9%) • EKG abnormal in 13/34 (38.2%): Sens 77%, Spec 76% for mortality • cTnI: Sens 33%, Spec 80%	• The assessment of myocardial dysfunction via serum cTnI levels and EKG changes in the early neonatal period provides significant predictive data for mortality in HIE • Conventional echocardiography (EF) may fail to detect early myocardial injury, possibly because left ventricular dysfunction is obscured by compensatory mechanisms in the first days of life
**Conventional Echocardiography (2D echo + Doppler): Therapeutic Hypothermia Era**				
Liu et al. (2007) [89]	70	Pulsatility index (PI), resistive index (RI), FS, Peak systolic flow velocity (PSFV), end-diastolic flow velocity (EDFV), time-averaged mean flow velocity (TMFV)	• While 100% of HIE patients exhibited LV diastolic dysfunction at the initial examination, systolic dysfunction was seen in 70% (28/40) of the patients • Diastolic dysfunction typically returned to normal within 3 days, whereas systolic dysfunction required at least 7 days to recover • There was a significant link between the degree of cardiac impairment and encephalopathy severity	• There is a significant correlation between myocardial function and cerebral hemodynamics in neonates with HIE • Cardiac dysfunction contributes to severe encephalopathy and the loss of cerebrovascular autoregulation, making it essential to preserve and treat myocardial function in these infants
Karaarslan et al. (2012) [90]	70	EF, FS, MPI	• EF and FS were within normal ranges for both the HIE and control groups, showing no significant difference • MPI was significantly higher in both severe and moderate HIE groups compared to controls during the first analysis (within 24 h of birth)	• Left and right ventricular MPI values are significantly elevated in babies with severe and moderate HIE shortly after birth • MPI ≥ 0.5 can be used as a discriminative additive criterion to help distinguish between moderate and severe HIE according to Sarnat grades
Aggarwal & Natarajan (2017) [91]	91	LVO, RVO, biventricular MPI, eccentricity index (systole/diastole), RV S/D ratio, PDA shunt direction	• Significantly elevated biventricular MPI, lower RVO and LVO, and pulmonary hypertension in HIE vs. controls • Infants who died had a significantly lower diastolic eccentricity index (EId) (0.77 vs. 0.83) compared to survivors	• Biventricular output and MPI are impaired in HIE. LV MPI was significantly lower in the HIE-TH group compared to the HIE-normothermia group (0.47 vs. 0.60, *p* < 0.01) • Eccentricity index in diastole is an echo predictor of mortality, reflecting pulmonary hypertension severity • Fractional shortening was not significantly different between HIE and controls
Shahidi et al. (2017) [92]	60	Systolic and diastolic function, pulmonary hypertension, and the presence of PDA	• Systolic dysfunction was present in about one-third of asphyxiated neonates and correlated with the severity of the asphyxia • Diastolic dysfunction was even more prevalent, detected in about half of the asphyxiated group	• The paper does not specify which exact echocardiographic parameters were used to define systolic and diastolic dysfunction • Birth asphyxia is strongly associated with both systolic and diastolic cardiac dysfunction, as well as pulmonary hypertension
Yoon et al. (2018) [93]	32	LVCO, EF, FS, descending aorta blood flow (DABF), upper body blood flow (UBBF), resistive index (RI)	• During TH, LVCO decreased by approximately 30% and DABF was significantly reduced compared to controls, while the RI of the descending aorta increased, indicating higher peripheral vascular resistance • UBBF remained unchanged, leading to a significantly increased UBBF/LVCO ratio (53.1% in patients vs. 31.9% in controls), which reflects a preferential cerebral redistribution of cardiac output • Urine output was significantly lower during TH and correlated positively with the DABF/LVCO ratio, suggesting that systemic organ hypoperfusion occurs during cooling	• TH is associated with decreased cardiac output and a shift in blood flow favoring the brain over systemic organs • This preferential redistribution, driven in part by increased peripheral vascular resistance, may lead to relative hypoperfusion of the kidneys and other organs • Exaggerated UBBF/LVCO ratio may serve as a marker for impaired cerebral autoregulation and more severe brain injury
Aggarwal et al. (2019) [94]	65	TAPSE, FAC, MPI, RV systolic to diastolic duration (S/D) ratio	• Infants requiring iNO/ECMO had significantly lower TAPSE and RV FAC, alongside significantly higher RV MPI and S/D ratios • The RV S/D ratio demonstrated the best predictive performance for severe PPHN, with an area under the ROC curve of 0.746 • At a cutoff of >1.45, the RV S/D ratio had a sensitivity of 76.9% and a negative predictive value of 78.6% for subsequent need for iNO or ECMO	• Neonates with HIE undergoing TH who progress to severe PPHN exhibit detectable RV dysfunction on early echocardiograms (before 24 h of age) • The RV S/D ratio is a simple and useful early marker for predicting the need for advanced therapies like iNO or ECMO
Sehgal et al. (2019) [95]	33	RVO, LVO, SV, RV FAC, TAPSE, FS, cTnT, TPV/RVETc (time to peak velocity/right ventricular ejection time corrected)	• At baseline (pre-cooling), 76% of infants had very low cardiac output (<150 mL/kg/min), with 97% demonstrating low TAPSE (<9 mm) • TAPSE and FAC were more sensitive markers of change than fractional shortening, which showed no significant pairwise changes across the phases	• Cardiac function in moderate-to-severe HIE is dynamic and evolves significantly across the phases of TH • TPV/RVETc showed significant changes across all phases and had a strong correlation with TAPSE (r^2^ = 0.68, *p* < 0.001) • FAC and TAPSE are sensitive to this evolution; FS is not
Giesinger et al. (2019) [16]	53	TAPSE, RV-FAC, LVEF, LV-FS, RVO, LVO	• TAPSE < 6 mm (sensitivity 78%, specificity 81%) and RV-FAC < 0.29 (sensitivity 83%, specificity 84%) were independent predictors of poor outcomes • LV EF and FS remained largely normal and were not associated with brain injury	• RV dysfunction at 24 h is independently associated with adverse outcome
Rodriguez MJ et al. (2020) [96]	57	TAPSE, RVO, RV stroke volume (SVRV), RV myocardial performance index (MPI), LVO, EF, FS, SVC flow, Cerebral blood flow (CBF) velocity	• Neonates with moderate-to-severe HIE undergoing TH exhibited predominant RV dysfunction (reduced TAPSE, RVO, and SVRV) during the first 24 h of life, whereas LV EF and FS remained largely preserved compared to controls • Significant positive correlation was found between cerebral blood flow (CBF) velocity (PSV and EDV) and RV metrics (RVO, SVRV, and TAPSE)	• Cerebral blood flow velocity and brain oxygenation correlate predominantly with RV function in cooled neonate • RV failure may be a direct contributor to altered brain perfusion and the potential aggravation of injury during TH
Vasiljevic et al. (2021) [97]	120	Transtricuspid pressure gradient (TRPG), FS, and left ventricular eccentricity index (LVEI)	• Asphyxiated neonates exhibited significantly higher TRPG and lower FS compared to controls, with non-survivors having a mean TRPG of 52.8 mmHg compared to 28.0 mmHg in survivors • cTnI and cTnT levels showed a significant correlation with echocardiographic parameters primarily by the third day of life, though they were found to be less sensitive than NT-proBNP for early outcome prediction	• There is a strong correlation between biochemical markers of injury and echocardiographic findings of cardiac dysfunction • Lactate is the most robust independent predictor of survival
Rasheed et al. (2024) [26]	166	LVEF, TR/MR, PDA, FS, left ventricular end-diastolic and systolic diameters (LVEDD/LVESD)	• The most common echocardiographic findings were pulmonary hypertension (50%), PDA (37.2%), and mitral regurgitation (6.6%) • Three independent determinants significantly increased the odds of myocardial dysfunction: severe asphyxia (aOR 5.01), the presence of PDA (aOR 5.11), and cesarean section delivery (aOR 2.65)	• Myocardial dysfunction is a common complication of perinatal asphyxia, and its risk is significantly higher in cases of severe asphyxia, delivery via cesarean section, or when a PDA is present • The study explicitly states that selective/total body hypothermia was unavailable at the institution (Important limitation)
Brunets et al. (2025) [98]	113	CO, FS, MAPSE, E/A, MPI, TDI	• MPI was significantly higher in the study group during TH, indicating impaired global function • TDI showed significantly lower E′ velocity and E′/A′ ratio during TH, which are markers of diastolic dysfunction	• Neonates undergoing TH exhibit more severe global cardiac impairment (higher MPI and lower E′/A′ ratios) compared to healthy controls
Thomas et al. (2025) [99]	40	Ventricular dimensions, systolic function	• The incidence of cardiac dysfunction increased with HIE severity: 25% in mild HIE, 81.8% in moderate HIE, and 100% in severe HIE • ECHO abnormalities occurred in 65% of neonates; moderate tricuspid regurgitation was the most frequent finding (20%)	• There is a strong association between the severity of HIE and myocardial involvement
**Tissue Doppler imaging (TDI)**				
Wei Y et al. (2009) [100]	92	EF, FS, SV, Peak systolic velocity of the anterior mitral valve leaflet (Sm wave)	• In the severe asphyxia group, LVEF and FS were significantly reduced at 24 h but recovered to normal ranges by 48–72 h • Sm wave was significantly lower in both mild and severe asphyxia groups compared to controls across all time points (24, 48, and 72 h)	• TDI-derived Sm is a more sensitive indicator of LV systolic dysfunction than conventional M-mode parameters (LVEF, FS, or SV) because it detects intrinsic myocardial movement that remains depressed even after global pumping indices appear to have recovered
Matter M et al. (2010) [101]	45	RV and LV Tei index, Peak systolic (Sm), early diastolic (Em), and late diastolic (Am) velocities	• LV and RV Tei indices were significantly higher (0.51 vs. 0.38 and 0.45 vs. 0.28, respectively) and Sm velocities were significantly lower in asphyxiated neonates compared to controls • Conventional markers (FS, Cardiac Index, and E/A ratios) showed no significant differences between the groups	• TDI is more sensitive than conventional echocardiography for the early detection of subclinical myocardial dysfunction following perinatal asphyxia
Nestaas et al. (2011) [102]	68	Longitudinal peak systolic strain (PSS) and peak systolic strain rate (PSSR) averaged across 18 myocardial segments, FS, EF	• PSS was significantly lower in asphyxiated neonates compared to healthy controls on the first three days of life (*p* < 0.001), PSSR was significantly lower only on day 2 (*p* = 0.0001) • Fractional shortening was nearly identical between groups	• Longitudinal strain and strain rate derived from TDI are more sensitive markers than fractional shortening for identifying reduced myocardial function in asphyxiated neonates
Nestaas et al. (2014) [103]	112	Peak systolic strain (PSS), peak systolic strain rate (PSSR), early diastolic strain rate (ESR) and strain rate in atrial systole (ASR), FS	• While the two HIE groups had broadly similar indices, PSSR was significantly lower in the TH-group than the NT-group on days 1 and 3, and ASR was significantly lower in the HT-group on day 1 • Following rewarming (Day 4), all strain rate indices improved significantly, reaching levels comparable to healthy 3-day-old infants	• TH appears to preserve the heart’s capacity for high-level contractility
Jiang et al. (2016) [104]	70	TDI, MPI, EF, FS	• Both left and right ventricular Tei indices were significantly higher in asphyxiated neonates • According to ROC curve analysis, the Tei indices demonstrated higher diagnostic value for identifying myocardial dysfunction than either cTnI or CK-MB	• The TDI-derived Tei index (MPI) is a more comprehensive marker of global cardiac dysfunction than conventional echocardiographic measures • Serum CK-MB correlates with TDI-derived Tei indices, supporting the complementary use of biochemical and imaging markers for early detection of myocardial injury after perinatal asphyxia
Nayak et al. (2021) [105]	60	EF, FS, LVEDD, LVESD, TAPSE, TDI, Tei index	• Significant differences were found in M-mode parameters (LVEDD, LVESD, FS) within the first 12 h of life • The Tei index was higher in asphyxiated neonates, indicating biventricular dysfunction, and was noted as a more sensitive marker than others	• Myocardial function assessment using a combination of cTnI and various echocardiographic techniques (especially TDI and the Tei index) is one of the most sensitive and specific method for detecting ischemic cardiac injury in asphyxiated newborns
Sobeih AA, et al. (2021) [106]	40	Mitral and tricuspid peak E and A wave velocities and E/A ratios, TDI, MPI	• Asphyxiated neonates had significantly more evidence of LV diastolic dysfunction and global myocardial impairment (indicated by higher Tei indices) compared to controls • TDI was more sensitive than conventional Doppler, detecting LV diastolic dysfunction in 17 out of 20 cases, whereas conventional pulsed wave Doppler only identified it in 12 out of 20 • Among nonsurvivors, the septal E′/A′ ratio was significantly lower, proving to be the most valuable prognostic indicators for predicting mortality in this group	• TDI is superior to conventional echocardiography for the early detection of cardiac dysfunction in neonates with perinatal asphyxia • RV diastolic dysfunction did not differ significantly between cases and controls by either conventional Doppler (*p* = 0.731) or TDI (*p* = 0.333)
Yajamanyam et al. (2022) [107]	40	FS, CO, TDI, MPI	• LV FS was not useful for detecting dysfunction as values were similar between cases and controls throughout all measurements • All TDI-derived myocardial velocities were significantly lower in cases on day 1, improved during TH, but LV indices remained consistently lower than controls even after rewarming	• In infants with moderate to severe HIE, LV dysfunction persists beyond the completion of TH into the first week of life, as demonstrated by TDI-derived parameters • LV function appears to be more affected than RV function in this population • Conventional LV FS was not sensitive enough to detect this dysfunction, highlighting the need for TDI-based assessment
Rodriguez MJ et al. (2022) [35]	36	Isovolumic contraction time (IVCT′), isovolumic relaxation time (IVRT′), ejection time (ET′), filling time (FT′)	• Neonates with HIE undergoing TH exhibited a distinct pattern of early diastolic dysfunction, characterized by globally prolonged IVRT′ in the left ventricle, right ventricle, and septum • Global dysfunction was evidenced by significantly increased MPI′ and t-IVT′ across both ventricles	• Perinatal HIE results in early diastolic dysfunction during therapeutic hypothermia, characterized by globally prolonged and slowed myocardial relaxation • Diastolic dysfunction can be subclinical during hypothermia-induced bradycardia, necessitating the use of TDI for early and non-invasive detection of myocardial damage
**Speckle Tracking Echocardiography**				
Sehgal et al. (2013) [108]	42	LV GLS, GLSR, LVO, FS	• Asphyxiated neonates demonstrated profoundly impaired LV GLS and significantly lower cardiac output • GLS correlated positively with cardiac output (r^2^ = 0.86, *p* < 0.001) and negatively with serum troponin (r^2^ = 0.64, *p* < 0.001) • Neonates who died had significantly lower global strain values compared to survivors (–9.7% vs. −12.8%)	• 2D speckle-derived GLS is a sensitive indicator of myocardial dysfunction in HIE
Breatnach et al. (2017) [109]	81	LV torsion (twist indexed to LV length), twist rate (LVTR), and untwist rate (LVUTR), GLS, GLSSR	• HIE neonates undergoing TH exhibited significantly depressed rotational mechanics • GLS and GLSSR were significantly impaired in the HIE cohort, while EF showed no significant difference	• Infants with HIE suffer from a global decrease in myocardial rotational and deformation mechanics • Rotational mechanics detect functional disruption that is undetected by EF
Altit et al. (2023) [110]	83	TAPSE, FAC, EF, peak longitudinal strain (pLS), peak longitudinal strain rate (pLSR)	• HIE infants showed decreased TAPSE, increased E/e′ ratios, and higher RV-MPI • The HIE group demonstrated significantly altered right and left ventricular deformation parameters (pLS and pLSR) via STE	• Neonates with HIE undergoing therapeutic hypothermia exhibit increased pulmonary pressures and altered biventricular systolic and diastolic performance
Jin JB et al. (2025) [38]	180	Left ventricular 4-chamber (LV 4Ch) strain, right ventricular 4-chamber (RV 4Ch) strain, and RV free wall strain (RV FWS)	• RV mechanics (RV 4Ch strain, RV FWS, RV FAC) were significantly impaired in the overall neonatal encephalopathy (NE) group • Severe NE was associated with significantly lower LV 4Ch strain and RV FAC compared to mild cases, correlated strongly with the need for chest compressions, epinephrine at delivery, and decreased survival to discharge • Traditional markers (LV EF, LV FS, and RV FAC) did not correlate with survival • LV 4Ch strain was not significantly different between the overall NE group and controls (−22.5% vs. −21.6%, *p* = 0.31)	• RV strain mechanics is impaired in neonates with NE, especially those with severe encephalopathy • Strain parameters might potentially be more sensitive and prognostic than traditional ejection fraction or fractional shortening
Hu et al. (2025) [86]	53	LVEF, SV, CO, GLS, global work index (GWI), global constructive work (GCW)	• In the severe asphyxia group, conventional markers like LVEF and CO recovered more quickly than MW parameters (GWI and GCW), suggesting MW reflects a more “microscopic” energy state of the myocardium • GWI at 24 h was negatively correlated with NT-proBNP levels, suggesting its early value in predicting cardiac impairment	• Myocardial work parameters (GWI and GCW) are valuable supplemental indicators for the dynamic monitoring of myocardial recovery after neonatal asphyxia • They provide a more sensitive assessment of the myocardial energy state and cardiac reserve than traditional echocardiographic measures
Biran V et al. (2025) [111]	70	Peak longitudinal strain (pLS), peak longitudinal strain rate (pLSR), and early diastolic strain rate (EDSR) for both ventricles	• At a median follow-up of 6.8 months, infants who had an initially abnormal aEEG on admission exhibited enhanced RV contractility—RV-pLSR, and RV free wall strain rate • Conventional metrics (TAPSE, FAC, EF, and RV/LV ratios) showed no significant differences between the groups at follow-up	• RV hypercontractility persists at 6–7 months post-NICU discharge in neonates with initial abnormal aEEG
Nunes et al. (2026) [112]	44	EF, FS, pulmonary artery systolic pressure (PASP), GLS, circumferential and radial strain, LV twist/torsion, and the Tei index	• LV systolic function (EF, SF, and GLS) remained comparable to the control group during therapeutic hypothermia, suggesting a potential cardioprotective effect • After rewarming, neonates exhibited a hyperdynamic circulatory state, with higher EF and SF than the control group • RV function was impaired, as evidenced by significantly worse RV GLS and higher PASP in the HIE group compared to controls	• Therapeutic hypothermia appears to be safe for cardiovascular performance and does not detrimentally impact LV systolic function • However, RV dysfunction is present in these neonates, primarily related to elevated pulmonary pressures, and RV GLS is a sensitive parameter for detecting this impairment

aEEG, amplitude-integrated electroencephalogram; CBF, cerebral blood flow; CO, cardiac output; EF, ejection fraction; FAC, fractional area change; FS, fractional shortening; GLS, global longitudinal strain; GLPSS, global longitudinal peak systolic strain; GLSSR, global longitudinal systolic strain rate; HIE, hypoxic–ischemic encephalopathy; HR, heart rate; LV, left ventricle; LVO, left ventricular output; LVCO, left ventricular cardiac output; MAP, mean arterial pressure; MPI, myocardial performance index; MRI, magnetic resonance imaging; NE, neonatal encephalopathy; pGCS, peak global circumferential strain; pGLS, peak global longitudinal strain; PI, pulsatility index; PSV, peak systolic velocity; RV, right ventricle; RVO, right ventricular output; sEI, systolic eccentricity index; STE, speckle-tracking echocardiography; SV, stroke volume; SVC, superior vena cava; TAPSE, tricuspid annular plane systolic excursion; TDI, tissue Doppler imaging; TH, therapeutic hypothermia.

**Table 6:** Association between markers of cardiac dysfunction and clinical outcomes in neonates with HIE, summarizing key studies, sample sizes, the cardiac parameter examined, the outcome measure, and the primary association identified. Outcomes are categorized as short-term (mortality prior to discharge, need for inotropic or extracorporeal support, and MRI-defined brain injury prior to discharge) and long-term (neurodevelopmental impairment at 18 months or beyond).

Author (Year)	Sample Size (n)	Cardiac Marker	Outcome Measure	Key Association
Giesinger et al. (2019) [16]	53	TAPSE, RV-FAC	Death or abnormal MRI	RV dysfunction independently predicts adverse composite outcome
Giesinger et al. (2022) [119]	46	TAPSE at 24 h	Neurodevelopmental outcomes at 18 months	TAPSE < 6 mm independently associated with adverse neurodevelopmental outcomes
Altit et al. (2023) [13]	53	Biventricular echo	Death or brain injury (by MRI)	• RV dysfunction (decreased TAPSE, *p* = 0.01) and lower blood pressure were associated with adverse outcome • LV function (by longitudinal strain and ejection fraction) was independently associated with adverse outcome • Elevated pulmonary artery pressure alone did not distinguish outcomes, but ventricular dysfunction did
Mohammad et al. (2017) [124]	221	Inotrope requirement	Death or abnormal MRI	Hemodynamic instability requiring inotropes in the first 72 h of life was associated with increased risk of death or brain injury detected by MRI
Liu et al. (2013) [61]	75	cTnI at 24 h	Neurodevelopmental outcomes	cTnI predicts neurodevelopmental outcomes at 18–22 months
Montaldo et al. (2018) [125]	64	Heart rate, RR and QTc intervals (EKG), and Superior Vena Cava (SVC) flow	Long-term neurological outcome (death or moderate/severe disability at 18–24 months)	Infants with adverse outcomes exhibited higher heart rates and significantly shorter RR and QTc intervals during therapeutic hypothermia. Furthermore, higher SVC flow pre-rewarming was significantly associated with higher odds of long-term adverse neurological outcomes
Kovacs et al. (2022) [123]	176	Duration of CV support (inotropes/vasopressors)	Death or abnormal MRI	24 h longer duration of cardiovascular support increased the odds for adverse outcome by 14% (short-term outcomes)
Shakir W, et al. (2022) [126]	171	CK-MB, EKG, Echo, Use of inotropes	Mortality within a 14-day follow-up period	Asphyxiated neonates with cardiovascular dysfunction (as indicated by the need for inotropes) had a mortality rate of 38.3%, compared to only 5.0% in those without such dysfunction (*p* < 0.0001)
Kilmartin et al. (2024) [39]	384	PPHN	Death and brain injury (by MRI)	PPHN associated with higher mortality and more brain injury
Kovacs et al. (2025) [40]	152	Duration of CV support (inotropes/vasopressors)	Death or neurodevelopmental impairment (NDI) defined as 70 on Bayley-II or 85 on Bayley-III	For every 24 h increase in duration of cardiovascular support, odds of adverse outcome increased by 12% (long term outcomes)
Lapointe et al. (2025) [29]	55	LV GLS (STE)	Brain injury (by MRI)	Severe initial aEEG patterns, higher gestational age, and increased LV contractility by strain analysis on DOL 2 were independently associated with brain injury. Standard echocardiographic measures (EF, FS, TAPSE) did not differ between groups

aEEG, amplitude-integrated electroencephalogram; CV, cardiovascular; EF, ejection fraction; FAC, fractional area change; GLS, global longitudinal strain; LV, left ventricle; MRI, magnetic resonance imaging; NDI, neurodevelopmental impairment; PPHN, persistent pulmonary hypertension of the newborn; RV, right ventricle; STE, speckle-tracking echocardiography; TAPSE, tricuspid annular plane systolic excursion.

## Data Availability

No new data were created or analyzed in this study. Data sharing is not applicable to this article.

## Abbreviations

The following abbreviations are used in this manuscript:

aPH	Acute pulmonary hypertension
BNP	B-type natriuretic peptide
CBF	Cerebral blood flow
CHD	Congenital heart disease
CK	Creatine kinase
CK-MB	Creatine kinase-MB isoenzyme
CO	Cardiac output
cTnI	Cardiac troponin I
cTnT	Cardiac troponin T
DABF	Descending aorta blood flow
EDV	End-diastolic volume
EF	Ejection fraction
EKG/ECG	Electrocardiogram
ESV	End-systolic volume
ET	Ejection time
FAC	Fractional area change
FS	Fractional shortening
FT	Filling time
FWS	Free wall strain
GLS	Global longitudinal strain
GWE	Global work efficiency
GWI	Global work index
HIE	Hypoxic–ischemic encephalopathy
HR	Heart rate
IVCT	Isovolumic contraction time
IVRT	Isovolumic relaxation time
LDH	Lactate dehydrogenase
LV	Left ventricle/left ventricular
LVCO	Left ventricular cardiac output
LVIDd	Left ventricular internal diameter at end-diastole
LVIDs	Left ventricular internal diameter at end-systole
LVEF	Left ventricular ejection fraction
LVO	Left ventricular output
MAP	Mean arterial pressure
MAPSE	Mitral annular plane systolic excursion
MCA	Middle cerebral artery
MFV	Mean flow velocity
MPI	Myocardial performance index
MRI	Magnetic resonance imaging
NDI	Neurodevelopmental impairment
NICU	Neonatal intensive care unit
NIRS	Near-infrared spectroscopy
NPV	Negative predictive value
NT-proBNP	N-terminal pro-B-type natriuretic peptide
PAAT	Pulmonary artery acceleration time
PCC	Population, Concept, Context
PDA	Patent ductus arteriosus
PFO	Patent foramen ovale
PH	Pulmonary hypertension
PI	Pulsatility index
POCUS	Point-of-care ultrasound
PPHN	Persistent pulmonary hypertension of the newborn
PPV	Positive predictive value
PSV	Peak systolic velocity
PVR	Pulmonary vascular resistance
RI	Resistive index
RV	Right ventricle/right ventricular
RV-FAC	Right ventricular fractional area change
RVET	Right ventricular ejection time
RVO	Right ventricular output
RVSP	Right ventricular systolic pressure
sEI	Systolic eccentricity index
Sm	Peak systolic myocardial velocity
SR	Strain rate
STE	Speckle-tracking echocardiography
SV	Stroke volume
SVR	Systemic vascular resistance
SVC	Superior vena cava
TAPSE	Tricuspid annular plane systolic excursion
TDI	Tissue Doppler imaging
TH	Therapeutic hypothermia
TMI	Transient myocardial ischemia
TnECHO	Targeted neonatal echocardiography
TR	Tricuspid regurgitation
VTI	Velocity–time integral
